# Disentangling Host-Microbiota Regulation of Lipid Secretion by Enterocytes: Insights from Commensals *Lactobacillus paracasei* and *Escherichia coli*

**DOI:** 10.1128/mBio.01493-18

**Published:** 2018-09-04

**Authors:** Asmaa Tazi, João Ricardo Araujo, Céline Mulet, Ellen T. Arena, Giulia Nigro, Thierry Pédron, Philippe J. Sansonetti

**Affiliations:** a Unité de Pathogénie Microbienne Moléculaire, INSERM Unité 1202, Institut Pasteur, Paris, France; b Collège de France, Paris, France; University of Edinburgh

**Keywords:** *Escherichia coli*, *Lactobacillus*, chylomicrons, high-fat diet, lipid metabolism, microbiota

## Abstract

The gut microbiota contributes to nutrients absorption and metabolism by enterocytes, but the molecular mechanisms involved remain poorly understood, and most conclusions are inferred from studies comparing germfree and conventional animals colonized with diverse bacterial species. We selected two model commensal microorganisms, Escherichia coli and Lactobacillus paracasei, to assess the role of the small-intestinal microbiota in modulating lipid absorption and metabolism by the epithelium. Using an integrated approach encompassing cellular and murine models and combining metabolic parameters measurement, lipid droplet imaging, and gene expression analysis, we demonstrated that under homeostatic conditions, L. paracasei promotes fat storage in enterocytes, whereas E. coli enhances lipid catabolism and reduces chylomicron circulating levels. The Akt/mammalian target of sirolimus (mTOR) pathway is inhibited by both bacterial species *in vitro*, indicating that several regulatory pathways are involved in the distinct intracellular lipid outcomes associated with each bacterial species. Moreover, soluble bacterial factors partially reproduce the effects observed with live microorganisms. However, reduction of chylomicron circulating levels in E. coli-colonized animals is lost under high-fat-diet conditions, whereas it is potentiated by L. paracasei colonization accompanied by resistance to hypercholesterolemia and excess body weight gain.

## Abstract (precis)

The specific contribution of each bacterial species within a complex microbiota to the regulation of host lipid metabolism remains largely unknown. Using two model commensal microorganisms, L. paracasei and E. coli, we demonstrated that both bacterial species impacted host lipid metabolism in a diet-dependent manner and, notably, that L. paracasei-colonized mice but not E. coli-colonized mice resisted high-fat-diet-induced body weight gain. In addition, we set up cellular models of fatty acid absorption and secretion by enterocytes cocultured with bacteria and showed that, *in vitro*, both L. paracasei and E. coli inhibited lipid secretion, through increased intracellular fat storage and enhanced lipid catabolism, respectively.

## INTRODUCTION

Over the last decade, the evolving knowledge of the human gut microbiota and its combined genomes, the factors influencing its composition, and its contribution to host metabolism have profoundly changed our vision of physiology, with mammals now seen as “holobionts” (1–4). Gut microbiota is indeed involved in a wide variety of physiological processes, including energy extraction from food through polysaccharide digestion, production of vitamins, and intestinal epithelium and immune system development, but also, beyond the intestine, in host behavior and global metabolism (3, 4). Common diseases such as diabetes, obesity, and atherosclerosis have also been correlated with imbalance and changes in composition of the corresponding microbiota (i.e., dysbiosis), in both humans and animal models, raising questions concerning causality links and molecular mechanisms (5).

Studies based on conventionalization of germfree (GF) animals have addressed these causality links and identified specific features of the gut microbiota contributing to host metabolism. For instance, microbial metabolites such as short-chain fatty acids (SCFA) not only are energy sources for the colonic epithelium but also affect the expression of several genes and eventually participate in hepatic *de novo* lipogenesis through the expression of several key enzymes, such as acetyl-coenzyme A (acetyl-CoA) carboxylase (ACC) and fatty acid synthase (FASN), and of their regulators, the carbohydrate responsive element binding protein (ChREBP) and sterol responsive element binding protein 1c (SREBP-1c) (6, 7). Importantly regarding the current work, the gut microbiota directly affects nutrients, especially fatty acids (FA), and cholesterol absorption by enterocytes (8, 9) and regulates key intestinal and metabolic functions such as insulin sensitivity, fat storage, and energy expenditure (10–12).

Hence, the evidence indicating the possibility of manipulating the gut microbiota with beneficial microbes to restore the physiological status of the host in cases of metabolic disorders is convincing. Indeed, in mice, the presence of some lactic acid bacterial species, mainly *Lactobacillus* spp. and *Bifidobacterium* spp., restores insulin sensitivity and reduces hypercholesterolemia and high-fat-diet (HFD)-induced obesity (13–16). While the data are encouraging, these effects remain strain specific and the molecular mechanisms are largely unknown. We thus addressed the host-microbiota cross talk affecting host lipid absorption and metabolism using two model microorganisms, namely, the Gram-positive species Lactobacillus paracasei and the Gram-negative species Escherichia coli, two representatives of the intestine’s early colonizers (13, 17, 18). E. coli colonizes up to 90% of the human population and remains the predominant aero/anaerobic species in the intestine. L. paracasei, as a component of dairy products, is permanently present in our intestinal environment and is associated with decreased fat storage in animal models of HFD-induced obesity (19, 20).

Here, using complementary approaches in animal and cellular models, we demonstrate that under normal diet conditions, L. paracasei increases fat storage in enterocytes’ cytosolic lipid droplets (LD), whereas E. coli enhances lipid catabolism, leading to decreased chylomicron circulating levels. Under conditions of maintenance on a HFD, reduction of circulating chylomicron levels is lost in E. coli-colonized animals but potentiated in L. paracasei-colonized animals, which eventually resist excess body weight gain and hypercholesterolemia.

## RESULTS

### L. paracasei and E. coli gut colonization in conventional mice following a microbiota-depleting treatment.

**FIG 1:**
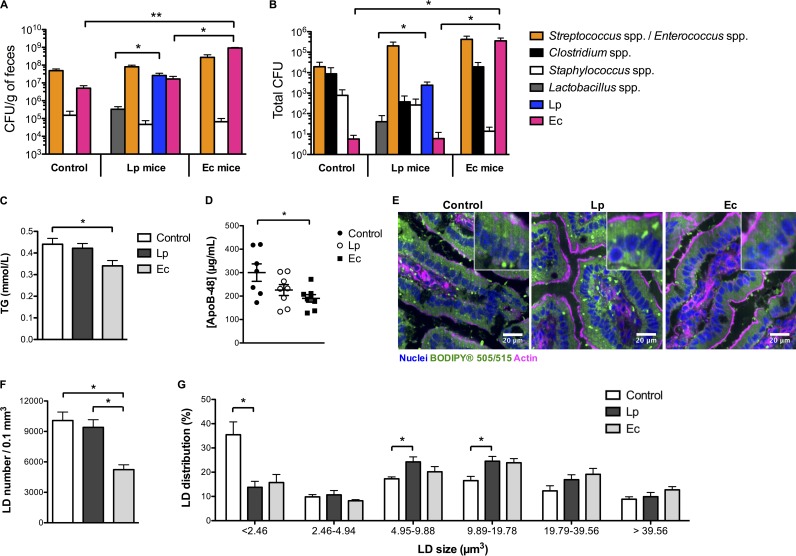
L. paracasei (Lp) and E. coli (Ec) colonization modulate intestinal lipid metabolism (*n* = 7 to 8 mice per group). Conventional mice were administered a microbiota-depleting antibiotic treatment before being gavaged with water (control), L. paracasei, or E. coli and maintained on a chow diet for 8 weeks. (A and B) Terminal microbiota composition assessed by conventional culture methods in the (A) feces and (B) ileum. (C and D) Terminal blood serum 1-h fasting levels of (C) TG and (D) ApoB-48 concentrations. (E) Representative confocal images (of 5 per condition) of jejunum sections stained with BODIPY showing intracellular lipid droplets. (F and G) Quantitative imaging analysis of intratissular intestinal LD using Imaris software, showing (F) mean LD number per condition and (G) LD distribution according to the size of the droplets. In panels A to D, F, and G, results are expressed as means ± standard errors of the means (SEM). Statistical significance is expressed relative to the control data unless otherwise specified. *, *P* < 0.05; **, *P* < 0.01 (one-way ANOVA).

To address the role of L. paracasei and E. coli in lipid absorption and secretion by enterocytes, and since the gastrointestinal tract of GF mice is considered immature and potentially associated with alterations of nutrient absorption (21), conventional specific-pathogen-free (SPF) mice were administered a gut microbiota-depleting treatment (22) to allow efficient L. paracasei and E. coli colonization of the intestine following gavage of mice with those bacterial species. Mice were maintained on a normal chow diet (CD) for 8 weeks, and stools were sampled for bacteriologic analysis. The number of CFU—approximately 5.10^9^ CFU/g of feces before treatment—was below the detection limit—5.10^2^ CFU/g of feces—at the end of the microbiota-depleting treatment for all the treated mice. Thereafter, conventional culture methods assessed a spontaneous gut recolonization by a microbiota mainly composed of streptococci and enterococci (Fig. 1A), although a larger pool of a microbial signature would be expected using metagenomics. In addition, L. paracasei and E. coli were maintained at approximately 1.10^7^ and 4.10^9^ CFU/g of feces in L. paracasei-gavaged mice (L. paracasei mice) and E. coli-gavaged mice (E. coli mice), respectively, throughout the experiment. End-course ileum colonization levels were 2.10^3^ and 3.10^5^ total CFU, respectively (Fig. 1B). Notably, L. paracasei colonization was accompanied by colonization by other *Lactobacillus* species and yet the colonization by the other *Lactobacillus* species occurred at a level 100-fold lower than that by L. paracasei, and E. coli, also found in the feces and ileum of control and L. paracasei mice, was highly enriched in E. coli-gavaged mice, i.e., by 1.10^2^ and 1.10^4^ times in the feces and ileum, respectively.

### E. coli colonization is accompanied by reduced chylomicron circulating levels under normal diet conditions.

Under normal diet conditions, no significant differences in either body weight curves or 1-h-fasting circulating cholesterol levels were observed among control, L. paracasei, and E. coli mice (data not shown), but E. coli mice showed a 20% reduction in circulating levels of triglycerides (TG) compared to control mice (Fig. 1C). Dietary lipids are absorbed by the apical brush border membrane of enterocytes and metabolized in TG and esterified cholesterol (EC) before storage in cytosolic lipid droplets (LD) or transfer to apolipoprotein B-48 (ApoB-48), which produces chylomicrons (23, 24). Chylomicrons are secreted into the lymph before reaching blood circulation and transport most of diet FA and cholesterol. Following their absorption, ApoB-48 returns to the liver as part of the chylomicron remnant, where it is degraded. Hence, serum levels of ApoB-48, known to be a unique marker for chylomicron secretion, were measured and found to be significantly (40%) reduced in E. coli mice (Fig. 1D), indicating that E. coli contributed to inhibition of lipid secretion by enterocytes and/or to increases in lipid clearance from the bloodstream.

To address the hypothesis of a decreased level of chylomicron biosynthesis in E. coli mice, levels of jejunal LD and prechylomicrons were compared among the different groups of mice using a neutral lipid staining fluorescent probe (BODIPY 505/515). Whereas no difference in enterocyte LD counts could be assessed between L. paracasei and control mice, a clear defect was observed in the small intestine of E. coli mice (Fig. 1E and F). No difference in food intake was observed between the groups of mice (3.2 ± 0.3 g, daily); thus, a lower level of fat ingestion in E. coli mice was unlikely, arguing for inhibition of lipid absorption or increased lipid consumption or both, eventually leading to diminished chylomicron biosynthesis. Conversely, L. paracasei colonization was accompanied by modifications of LD size distributions, with a trend toward LD of larger size (Fig. 1G), suggesting modifications of intracellular lipid metabolism and increased lipid storage.

### Decreased intestinal PPAR-regulated gene expression following L. paracasei and E. coli colonization.

**FIG 2:**
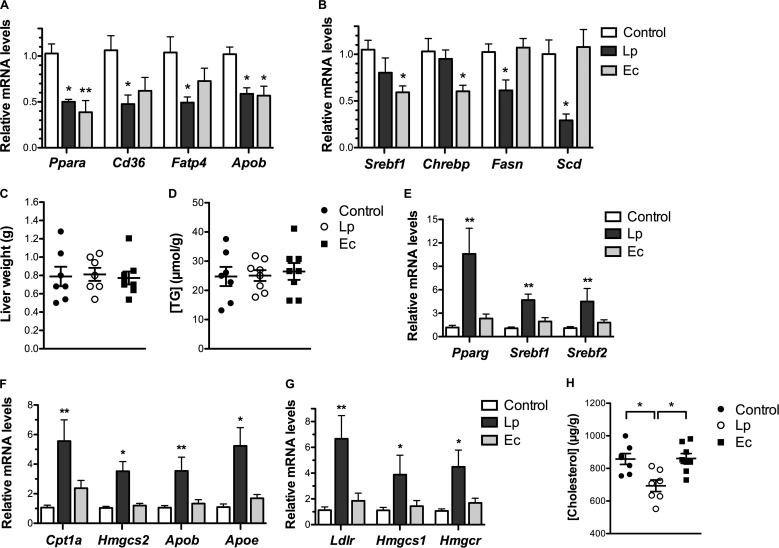
Intestinal and hepatic reprogramming of gene expression following L. paracasei (Lp) and E. coli (Ec) colonization (*n* = 7 to 8 mice per group). Conventional mice were administered a microbiota-depleting antibiotic treatment before being gavaged with water (control), L. paracasei or E. coli and maintained on a chow diet for 8 weeks. (A and B) terminal intestinal expression levels assessed by RT-qPCR of (A) PPAR-controlled genes and (B) lipogenic genes. (C) Liver weight. (D) Liver TG content expressed in micromoles per gram of liver weight. (E to G) Terminal hepatic expression levels assessed by RT-qPCR of (E) regulators of lipid metabolism, (F) PPAR targets, and (G) SREBP-2 targets involved in cholesterol homeostasis. (H) Liver total cholesterol content expressed in micrograms per gram of liver weight. In panels A and B and panels E to G, results are normalized to the *Actin* gene and expressed as means of fold change relative to the control ± SEM. In panels C, D, and H, results are expressed as means ± SEM. Statistical significance is expressed relative to the control unless otherwise specified. *, *P* < 0.05; **, *P* < 0.01 (one-way ANOVA).

To decipher the molecular mechanisms behind the reduction of chylomicron circulating levels in E. coli mice and the trend toward a modification of LD distribution in L. paracasei mice, we analyzed intestinal expression of key genes involved in lipid metabolism (see Fig. S1 and Table S1 in the supplemental material). L. paracasei colonization was associated with a corresponding ~2-fold transcriptional decrease in the level of peroxisome proliferator-activated receptor (PPAR) alpha (*Ppara*), of PPAR targets involved in FA uptake (*Cd36*, *Fatp4*), and of the *Apob* gene (Fig. 2A). Similar changes in *Ppara* and *Apob* expression were observed in E. coli mice.

10.1128/mBio.01493-18.1 FIG S1  Simplified schematic view of lipid absorption and metabolism in enterocytes. The main steps of intracellular lipid metabolism are represented together with the main transcriptional regulators in brackets (1). Dietary triglycerides (TG) are emulsified by bile acids and digested by the pancreatic lipase in fatty acids (FA), monoacylglycerides (MAG), and diacylglycerides (DAG), which are taken up by the brush border and absorbed by passive diffusion or by the fatty acid translocase FAT/CD36 and the scavenger receptor B1 SR-B1. Dietary free cholesterol is absorbed by the cholesterol transporter NPC1L1 (2). Cholesterol absorption and metabolism are mainly regulated by SREBP-2, whose activity depends on the intracellular cholesterol content (3). FA, MAG, and DAG are reesterified in TG (4). Absorbed FA can be degraded through FA beta-oxidation and FA oxidation, and TG and chylomicrons synthesis is regulated by the three types of PPARs (PPARα, PPARβ/δ, and PPARγ) upon activation by their ligands, mainly polyunsaturated FA (5). FA *de novo* biosynthesis depends on LXR, ChREBP, and SREBP-1c, which are regulated by insulin signaling and glucose (6). TG are taken up by the microsomal-triglyceride transfer protein (MTTP) and incorporated with cholesterol, esterified cholesterol, and apolipoproteins in prechylomicrons, which are eventually secreted in the mesenteric lymph as chylomicrons or stored in cytosolic lipid droplets. Download FIG S1, TIF file, 0.6 MB. Copyright © 2018 Tazi et al. 2018 Tazi et al. This content is distributed under the terms of the Creative Commons Attribution 4.0 International license.

10.1128/mBio.01493-18.5 TABLE S1  Small intestine gene expression levels assessed by RT-qPCR in mice colonized with L. paracasei or E. coli in chow diet. Download TABLE S1, DOCX file, 0.02 MB. Copyright © 2018 Tazi et al. 2018 Tazi et al. This content is distributed under the terms of the Creative Commons Attribution 4.0 International license.

The 3 different types of PPAR, PPARα, PPARβ/δ (*Ppard*), and PPARγ (*Pparg*), critically contribute to the regulation of lipid metabolism upon activation by their ligands, mainly polyunsaturated FA (25). Decreased intestinal expression of PPAR-controlled genes suggested lower intracellular levels of free FA (FFA) in L. paracasei and E. coli mice, resulting from decreased absorption or from increased incorporation in TG or from increased FA oxidation or a combination of those factors.

### Intestinal downregulation of lipogenic genes and of lipogenic gene regulators following L. paracasei and E. coli colonization, respectively.

Apart from PPAR, SREBP-1c (*Srebf1*) and ChREBP (*Chrebp*) represent other critical regulators of lipid metabolism (7). Their targets involved in FA *de novo* biosynthesis (*Fasn*, *Scd*) were specifically downregulated in the small intestine of L. paracasei mice (Fig. 2B), consistent with increased intracellular lipid content. Thus, L. paracasei colonization appeared (i) to inhibit dietary fat absorption, as suggested by the downregulation of FA transporters and of the cholesterol transporter Niemann-Pick C1-L1 (*Npc1l1*) (Table S1), and (ii) to increase dietary fat incorporation and storage in cytosolic LD, as assessed by imaging showing a shift toward LD of larger size and decreased FA biosynthesis and *Apob* gene expression.

On the other hand, despite the decreased intestinal expression of *Srebf1* and *Chrebp*, none of their target genes was specifically reduced in E. coli mice (Fig. 2B). Regulation mediated by SREBP-1c and CHREBP is primarily posttranslational and driven by insulin and intracellular glucose levels, respectively. Thus, noncoordinated expression of *Srebf1* and *Chrebp* and their targets is possible. Nevertheless, the modifications of intestinal gene expression observed in E. coli mice, i.e., the downregulation of PPAR targets associated with *Srebf1* downregulation, resembled the consequences of mammalian target of sirolimus (mTOR) inhibition encountered during fasted states (26). Together with LD imaging showing a dramatic decrease of intestinal LD levels, these data suggested an increase in FA beta-oxidation in E. coli mice eventually leading to diminished chylomicron biosynthesis.

### Reprogramming of hepatic lipid metabolism following L. paracasei and E. coli colonization.

Although L. paracasei colonization and E. coli colonization were associated neither with major alterations of circulating TG levels nor with modifications of liver weight and TG content (Fig. 2C and D), colonization with both bacterial species led to important modifications of hepatic gene expression (Fig. 2E to G; see also Table S2 in the supplemental material). In particular, L. paracasei colonization induced specific modifications resembling mTORC1 pathway activation, including significant 4-to-10-fold overexpression of *Srebf1*, *Pparg* (Fig. 2E), and PPAR downstream targets (Fig. 2F), suggesting increased hepatic TG uptake, lipid oxidation, and secretion of lipoproteins (27). It also induced overexpression of *Srebf2*, which is, in contrast to SREBP-1c, solely regulated by intracellular cholesterol content, and of SREBP-2 targets (Fig. 2E and 2G), supporting decreased hepatic cholesterol levels and enhanced cholesterol biosynthesis and uptake (7). Accordingly, the total cholesterol content of the liver was diminished in L. paracasei mice in comparison to control and E. coli mice (Fig. 2H). Thus, although it had no impact on circulating lipid levels, L. paracasei colonization appeared to specifically reprogram both intestinal and hepatic gene expression, underlining its role in the regulation of lipid metabolism. On the other hand, E. coli colonization was mostly characterized by 4-fold overexpression of ATP-citrate lyase (*Acly*) (Table S2), which links carbohydrate metabolism and production of FA by converting citrate in acetyl-CoA, emphasizing the importance of carbohydrate metabolism regulation in E. coli mice.

10.1128/mBio.01493-18.6 TABLE S2  Hepatic gene expression levels assessed by RT-qPCR in mice colonized with L. paracasei or E. coli in chow diet. Download TABLE S2, DOCX file, 0.1 MB. Copyright © 2018 Tazi et al. 2018 Tazi et al. This content is distributed under the terms of the Creative Commons Attribution 4.0 International license.

### Enterocytes cocultured with L. paracasei or E. coli exhibit decreased extracellular TG concentrations.

To confirm our *in vivo* observations and to gain better insight into the direct bacterial regulation of enterocytes’ lipid metabolism, we used the noncancerous immortalized murine enterocyte cell line m-ICcl2 (28), which was cultured to full differentiation and polarity on transwell inserts in order to measure TG levels, gene expression levels, and LD biogenesis following 16 h of L. paracasei or E. coli coculture. Compared to nonexposed cells (control), the cocultures did not cause significant cytotoxicity (Fig. S2).

10.1128/mBio.01493-18.2 FIG S2  L. paracasei and E. coli modulate BODIPY C_12_ incorporation in intracellular LD. m-ICcl2 cells were polarized on transwell inserts for 14 to 21 days before infection of the upper compartment with bacteria (MOI of 100) was performed. (A) Supernatants were recovered after 16 h of stimulation, and cytotoxicity was assessed by measurement of LDH release. Control: non-exposed cells. A total of ≥3 experiments were performed in triplicate (one-way ANOVA). (D to G) Following 16 h of stimulation, the upper-compartment cellular medium of the culture chamber was replaced by fresh medium containing BODIPY C_12_ fluorescent lipid micelles and was then replaced by regular cell culture medium following 10 min of incubation. Supernatants were sampled, and cells were fixed for staining at the indicated time points. Control: non-exposed cells. (B and C) Representative confocal microscopy images (B) 1 h and (C) 2 h after the addition of lipid micelles, showing incorporation of BODIPY C_12_ in intracellular LD in green. Scale bar, 20 µm. (D to G) Quantitative image analysis of LD by the use of Imaris software following the addition of lipid micelles. Data represent the distribution of LD according to droplet size 1 h (D and E) and 2 h (F and G) after the addition of lipid micelles. Panels E and G are enlarged views of panels D and F, respectively, showing the proportions of LD that were ≥4.95 µm^3^ in size. *, *P* < 0.05; **, *P* < 0.01; ***, *P* < 0.001 (two-way ANOVA). A total of 3 experiments were performed in duplicate. Download FIG S2, TIF file, 1.3 MB. Copyright © 2018 Tazi et al. 2018 Tazi et al. This content is distributed under the terms of the Creative Commons Attribution 4.0 International license.

**FIG 3:**
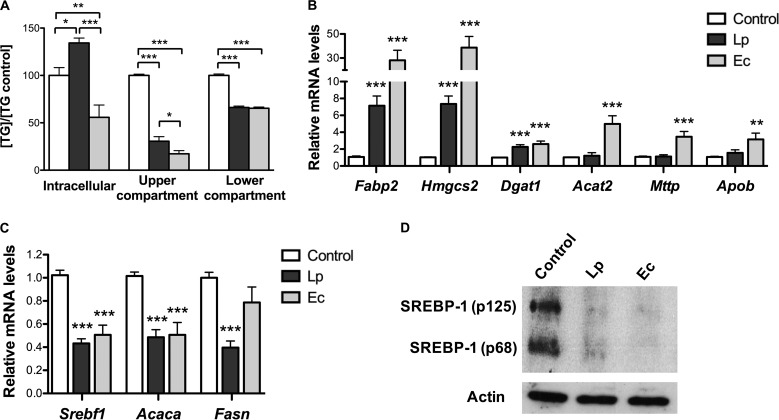
L. paracasei (Lp) and E. coli (Ec) impair TG secretion and lipid metabolism of cultured enterocytes. m-ICcl2 cells were polarized on transwell inserts for 14 to 21 days before infection of the upper compartment with bacteria (multiplicity of infection [MOI] 100). Supernatants and cell lysates were recovered after 16 h of infection. Control: noninfected cells. (A) Intracellular and supernatant TG levels. Results are normalized to control data and expressed as means ± SEM. (B and C) mRNA levels of genes involved in host lipid metabolism, including (B) PPAR-controlled genes and (C) SREBP-1c targets, assessed by RT-qPCR using *Actin* as reporter gene. Results are expressed as means of fold change ± SEM relative to the control. (D) Western blot analysis of SREBP-1 (3 experiments were performed in duplicate). Representative sample blots of total cell lysates show the cytoplasmic precursor (p125) and the nuclear form (p68) of SREBP-1 protein. (A to C) Statistical significance is expressed relative to the control unless otherwise specified. *, *P* < 0.05; **, *P* < 0.01; ***, *P* < 0.001 (one-way ANOVA). A total of ≥3 experiments were performed in triplicate.

L. paracasei and E. coli induced a significant decrease in TG levels both in the upper compartment (apical absorptive side) and in the lower compartment (basal secretory side) of the cell chamber (Fig. 3A). However, TG intracellular levels were differentially modulated by each bacterial species, i.e., were increased when cells were cultured with L. paracasei and reduced when they were cultured with E. coli, supporting increased lipid absorption and storage in cells cultured with L. paracasei and enhanced TG and FA consumption in the presence of E. coli.

### Reprogramming of enterocytes’ lipid metabolism by L. paracasei and E. coli.

To elucidate the molecular mechanisms behind the modifications in TG concentrations induced by each bacterial species, we analyzed cellular gene expression after 16 h of cell-bacterium coculture (Table S3). In contrast to intestinal gene expression of L. paracasei mice, PPAR-regulated gene expression was not inhibited in enterocytes cocultured with L. paracasei, and PPAR targets fatty acid binding protein 2 (*Fabp2*), which prevents FA transport back to the lumen, and 3-hydroxy-3-methylglutaryl-CoA (HMGC) synthase 2 (*Hmgcs2*), involved in ketogenesis, showed ~8-fold overexpression (Fig. 3B). Conversely, SREBP-1c targets involved in FA *de novo* biosynthesis were downregulated 2-fold (Fig. 3C), as in the small intestine of L. paracasei mice, and both the inactive cytoplasmic and active nuclear forms of SREBP-1 were barely detectable (Fig. 3D).

10.1128/mBio.01493-18.7 TABLE S3  m-ICcl2 gene expression levels assessed by RT-qPCR following 16 h of infection with L. paracasei or E. coli. Download TABLE S3, DOCX file, 0.02 MB. Copyright © 2018 Tazi et al. 2018 Tazi et al. This content is distributed under the terms of the Creative Commons Attribution 4.0 International license.

Consistent with intestinal gene expression of E. coli mice, inhibition of SREBP-1c was also observed in E. coli cocultured enterocytes but not, as expected with regard to the dramatic decrease in SREBP-1 protein levels, of its target *Fasn* (Fig. 3C and D). In parallel, E. coli cocultured enterocytes showed moderate 2-to-4-fold increases in the levels of PPAR targets involved in TG and lipoprotein biosynthesis and ~30-fold overexpression of *Fabp2* and *Hmgcs2* (Fig. 2B). Taken together, these results are consistent with increased FA uptake and FA oxidation and decreased TG levels in the extracellular upper compartment induced by both L. paracasei and E. coli.

### L. paracasei and E. coli inhibit Akt/mTORC1 signaling in enterocytes.

**FIG 4:**
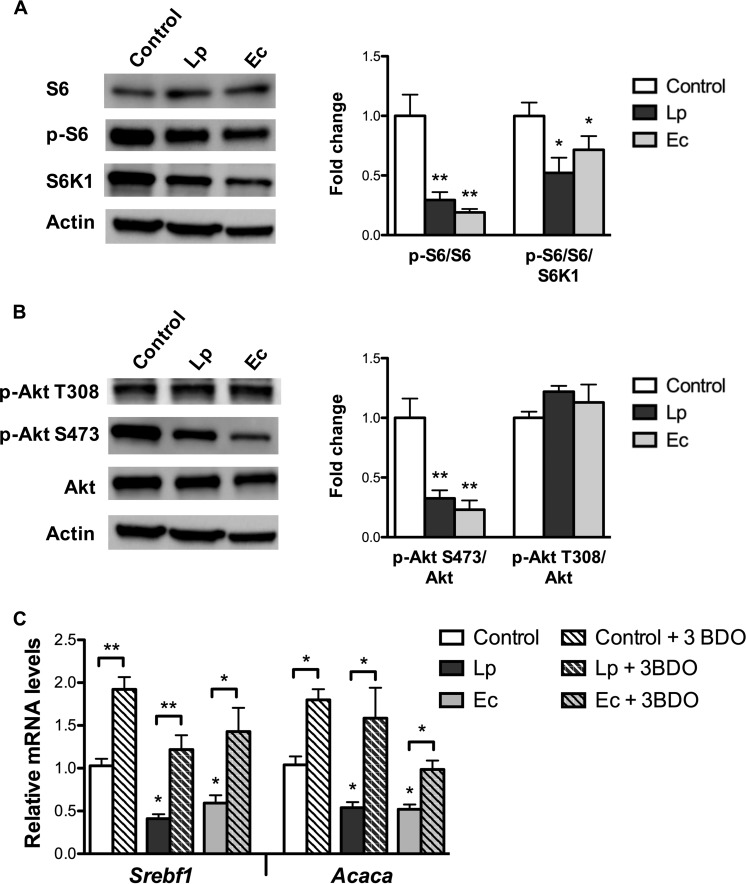
L. paracasei (Lp) and E. coli (Ec) inhibit the Akt/mTOR pathway in cultured enterocytes. m-ICcl2 cells were polarized on transwell inserts for 14 to 21 days before infection of the upper compartment with bacteria (MOI of 100); cell lysates were recovered following 16 h of infection. Control: non-exposed cells. (A and B) Western blot analysis of (A) S6 signaling and (B) Akt signaling. Cell lysates were subjected to Western blot analysis for (A) phosphorylated and total endogenous S6 and S6K1 and for (B) phosphorylated and total endogenous Akt. Representative sample blots and quantifications of S6 and Akt phosphorylation levels are shown. Values are presented as ratios between levels of phosphorylated protein S6 (A) or Akt and total endogenous protein (B), normalized to control cells. (C) mRNA levels of *Srebf1* and *Acaca* assessed by RT-qPCR using the *Actin* gene as the reporter gene following 16 h of coincubation with bacteria and with the mTOR activator 3-BDO. Results are expressed as means of fold change ± SEM. Statistical significance is expressed relative to the control unless otherwise specified. *, *P* < 0.05; **, *P* < 0.01 (one-way ANOVA). A total of 3 experiments were performed in duplicate.

Gene expression reprogramming of L. paracasei and E. coli cocultured enterocytes indicated a shift toward a “fasted” phenotype similar to that observed in the small intestine of E. coli mice, including decreased *Srebf1* mRNA levels, decreased SREBP-1c signaling, and increased beta-oxidation, features resembling results of mTOR pathway inhibition (26). The mTOR kinase nucleates two protein complexes, mTOR complex 1 (mTORC1) and mTORC2. Once activated, mTORC1 directly phosphorylates ribosomal protein S6 kinase 1 (S6K1), which in turn phosphorylates a number of targets, including ribosomal protein S6. Decreased ratio between levels of phosphorylated protein S6 (p-S6) and total protein S6 were observed in cells cocultured with both L. paracasei and E. coli (Fig. 4A), confirming inhibition of S6K1 and thus of mTORC1 activity.

mTORC1 is itself a downstream target of Akt (also known as protein kinase B), which responds to growth factors. Phosphorylation of Akt on Thr-308 and Ser-473 is required for full activity. Decreased protein levels of phosphorylated Akt on Ser-473 were also observed in cells cocultured with L. paracasei and E. coli (Fig. 4B), implying that mTORC1 pathway inhibition by both bacterial species involves factors acting upstream of Akt.

In addition, cell incubation with the direct mTOR activator 3-benzyl-5-[(2-nitrophenoxy)methyl]-dihydrofuran-2(3H)-one (3BDO) (29) increased the mRNA levels of *Srebf1* and of its target *Acaca* in nonstimulated enterocytes and in enterocytes cocultured with L. paracasei and E. coli (Fig. 4C), confirming that mTOR inhibition was critical for *Srebf1* downregulation by L. paracasei and E. coli.

### L. paracasei and E. coli impair lipid secretion by cultured enterocytes through increased storage and catabolism, respectively.

**FIG 5:**
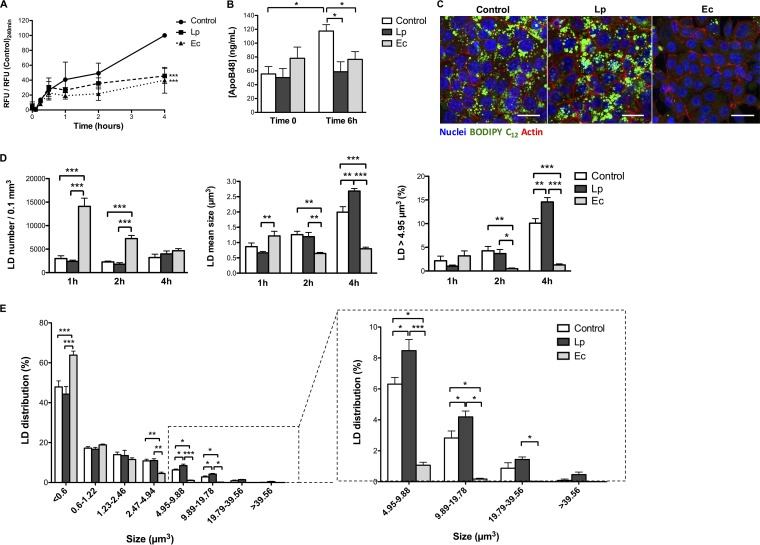
L. paracasei (Lp) and E. coli (Ec) inhibit chylomicron secretion by cultured enterocytes through increased lipid storage and catabolism, respectively. m-ICcl2 cells were polarized on transwell inserts for 14 to 21 days before infection of the upper compartment with bacteria (MOI of 100). Following 16 h of stimulation, the upper compartment cellular medium was replaced by fresh medium containing BODIPY C_12_ fluorescent lipid micelles for 10 min, then replaced by regular cell culture medium (time zero). Supernatants were sampled and cells were fixed for staining at the indicated time points. Control: non-exposed cells. (A) Secretion kinetics of BODIPY C_12_. The lower compartment cellular medium of the culture chamber was sampled at the indicated time points and the fluorescence measured. Values are expressed as means ± SEM of ratios between relative fluorescence units (RFU) of stimulated cells and RFU of control cells 4 h after the addition of lipid micelles. (B) ApoB-48 concentration in the lower compartment cell medium prior to and 6 h after the addition of lipid micelles. Results are expressed as means ± SEM. (C) Representative confocal microscopy images 4 h after the addition of lipid micelles showing incorporation of BODIPY C_12_ in intracellular LD (in green). Scale bar, 20 µm. (D and E) Quantitative imaging analysis of LD following the addition of lipid micelles using Imaris software. (D) LD numbers and mean sizes and proportions of LD larger than 4.95 µm^3^ at the indicated time points. (E) Distribution of lipid droplets according to size 4 h after the addition of lipid micelles. (A and B) A total of ≥3 experiments were performed in triplicate. (C to E) A total of 3 experiments were performed in duplicate, representing a total of 18 images per condition. *, *P* < 0.05; **, *P* < 0.01; ***, *P* < 0.001 (panels A and B, one-way ANOVA; panels C and D, two-way ANOVA).

Yet biochemical measurements and gene expression analyses argued for distinct lipid outcomes induced by L. paracasei and E. coli, i.e., increased storage and catabolism, respectively. To confirm this hypothesis, FA fate was monitored using lipid micelles containing a fluorescent long-chain FA (LCFA) analogue, BODIPY C_12_, added to the medium of the upper cellular compartment of cells precultured for 16 h with bacteria. Lipoprotein secretion was reflected by fluorescence levels in the medium of the lower compartment and steadily increased over time in control cells (Fig. 5A). In contrast, preculture of enterocytes with either L. paracasei or E. coli resulted in a 60% fluorescence reduction in the lower compartment 4 h after the addition of lipid micelles compared to control results. Inhibition of chylomicron secretion was further confirmed by ApoB-48 measurements showing a 40% decrease 6 h after the addition of lipid micelles to cells precultured with L. paracasei or E. coli compared to nonexposed controls (Fig. 5B).

Imaging of intracellular LD resulting from the metabolism of fluorescent FA molecules into TG and subsequent incorporation in cytosolic droplets or prechylomicrons showed that cocultures of L. paracasei and enterocytes induced an increase in LD size, whereas LD appeared massively reduced in size to the point of being barely visible in cells cultured with E. coli (Fig. 5C). Quantitative image analysis showed that in control cells and in cells cultured with L. paracasei, LD increased in size over time, as did the proportion of large LD (>4.95 µm^3^), with the increases reaching 10% and 15%, respectively (Fig. 5D; see also Fig. S2). Conversely, cells cultured with E. coli showed a larger amount of LD, which decreased over time in both number and size, and large LD never represented more than 3% of all LD, indicating increased FA uptake and consumption under this condition. Thus, 4 h after the addition of lipid micelles to the culture medium, the vast majority of LD in E. coli cocultured cells was represented by micro-LD (<0.6 µm^3^), whereas L. paracasei cocultured cells contained a higher proportion of large LD than control cells (Fig. 5D and E), supporting the idea of decreased chylomicron secretion due to L. paracasei-induced higher levels of lipid storage and E. coli-induced lipid catabolism.

### Conditioned medium from enterocytes—L. paracasei coculture and E. coli soluble factors partially reproduce live bacterium-mediated alterations of enterocytes’ lipid metabolism.

**FIG 6:**
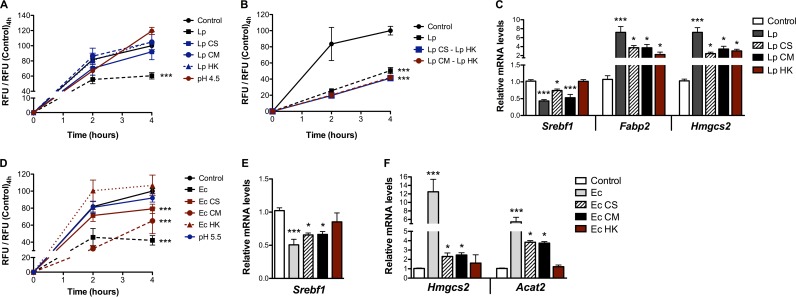
Soluble bacterial factors partially reproduce lipid metabolism modifications induced by live bacteria on cultured enterocytes. m-ICcl2 cells were polarized on transwell inserts for 14 to 21 days before infection of the upper compartment with bacteria (MOI of 100) or exposure to bacterial culture supernatants (CS), conditioned media (CM), heat-killed (HK) bacteria, or acidic pH. Following 16 h of incubation, the fluorescent lipid secretion assay was performed as described for Fig. 5 or cell lysates were collected for gene expression analysis. (A and B) Secretion kinetics of BODIPY C_12_. (C) mRNA levels of genes involved in host lipid metabolism under corresponding L. paracasei (Lp) conditions. (D) Secretion kinetics of BODIPY C_12_. (E and F) mRNA levels of genes involved in host lipid metabolism under corresponding E. coli (Ec) conditions. In panels A, B, and D, the lower compartment cellular medium of the culture chamber was sampled at the indicated time points and the levels of fluorescence were measured. Values are expressed as means ± SEM of ratios between RFU of exposed cells and RFU of control cells 4 h after the addition of lipid micelles. In panels C, E, and F, mRNA levels assessed by RT-qPCR using the *Actin* gene as the reporter gene are shown. Results are expressed as means of fold change ± SEM relative to the control. *, *P* < 0.05; **, *P* < 0.01; ***, *P* < 0.001 (one-way ANOVA). A total of ≥3 experiments were performed in triplicate.

Looking for bacterial factors responsible for the modifications of lipid processing induced by L. paracasei and E. coli, we analyzed enterocytes’ responses following 16 h of exposure to heat-killed (HK) bacteria, bacterial culture supernatants (CS), supernatants of cells cocultured with bacteria (referred to here as conditioned media [CM]), and artificially acidified medium (equivalent to acidification resulting from L. paracasei and E. coli growth, i.e., pH 4.5 and pH 5.5, respectively). None of the respective L. paracasei-based media affected lipoprotein secretion by enterocytes, as assessed by the fluorescent lipid secretion assay (Fig. 6A), nor did they recapitulate the modifications of TG levels observed in cells cocultured with bacteria (Table S4). However, cell stimulation with L. paracasei CS or L. paracasei CM combined with HK bacteria inhibited lipid secretion at a level similar to that seen with live bacteria (Fig. 6B) and the distinct L. paracasei-based media partially reproduced the alterations of mRNA levels induced by live bacteria (Fig. 6C). Interestingly, *Fabp2* which has been shown to reduce apolipoprotein biogenesis and chylomicron secretion (30), was overexpressed by L. paracasei supernatants and by HK L. paracasei to levels similar to those seen with live bacteria. Eventually, both the bacterial structural components and the bacterial soluble factors were shown to be necessary for L. paracasei-mediated alterations of enterocytes’ lipid metabolism.

10.1128/mBio.01493-18.8 TABLE S4  Impact of bacterial factors and acidic pH on m-ICcl2 enterocytes. Download TABLE S4, DOCX file, 0.1 MB. Copyright © 2018 Tazi et al. 2018 Tazi et al. This content is distributed under the terms of the Creative Commons Attribution 4.0 International license.

On the other hand, the fluorescent lipid secretion assay showed reduced fluorescence levels in the lower compartment following cell exposure to both E. coli CS and CM, indicating inhibition of lipoprotein secretion under these conditions (Fig. 6D). As culture supernatants, HK bacteria, and acidic pH induced similar changes in cytokine gene expression levels (Fig. S3), the possibility that mere inflammatory responses were responsible could be excluded. However, although none of the E. coli-based media could reproduce the modifications of TG levels and the overall gene expression pattern observed with live bacteria, they all differentially affected the expression of key genes involved in lipid metabolism (Table S4). Notably, E. coli CS and CM inhibited *Srebf1* expression (Fig. 6E), leading to overexpression of *Acat2* and *Hmgcs2*, which are involved in FA degradation and ketogenesis (Fig. 6F). Hence, E. coli CS-induced and CM-induced alterations of enterocyte gene expression resembled those seen with live bacteria, suggesting that E. coli soluble factors were capable of inhibiting chylomicron secretion following exposure to lipid micelles through enterocyte reprogramming toward enhanced beta-oxidation.

10.1128/mBio.01493-18.3 FIG S3  Expression levels of cytokine genes in enterocytes stimulated with L. paracasei-based and E. coli-based media. m-ICcl2 cells were polarized on transwell inserts for 14 to 21 days before infection of the upper compartment was performed with bacteria (MOI of 100) or exposure to bacterial culture supernatants (CS), conditioned media (CM), heat-killed (HK) bacteria, or acidic pH. Following 16 h of incubation, total RNAs were extracted for gene expression analysis of cytokines under (A) L. paracasei-based and (B) E. coli-based conditions using RT-qPCR with the actin gene used as a reporter gene. Results are expressed as means of fold change ± SEM relative to the control data. *, *P* < 0.05; **, *P* < 0.01; ***, *P* < 0.001 (one-way ANOVA). A total of ≥3 experiments were performed in triplicate. Download FIG S3, TIF file, 1.5 MB. Copyright © 2018 Tazi et al. 2018 Tazi et al. This content is distributed under the terms of the Creative Commons Attribution 4.0 International license.

### Conventional mice colonized with L. paracasei resist HFD-induced body weight gain and hypercholesterolemia.

**FIG 7:**
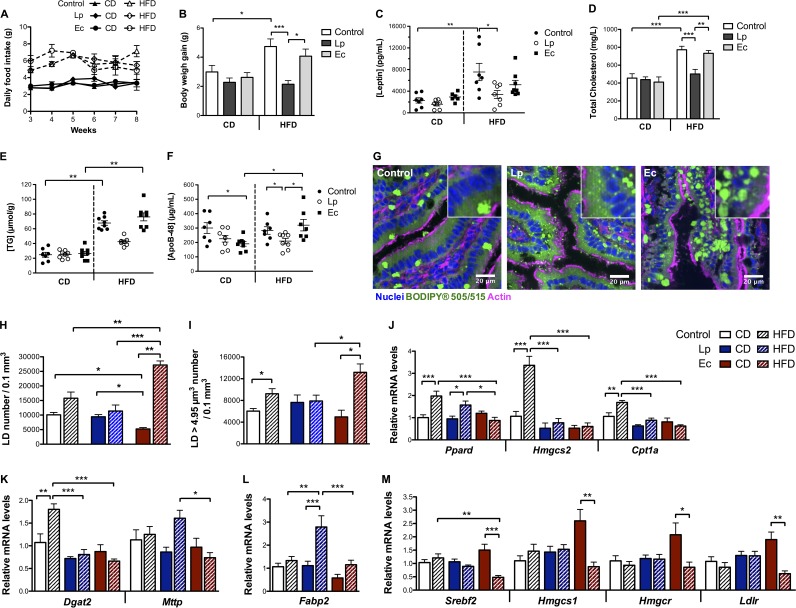
L. paracasei (Lp) and E. coli (Ec) colonization affect lipid secretion by enterocytes and the host’s response to a HFD (*n* = 7 to 8 mice per group). Conventional mice were administered a microbiota-depleting antibiotic treatment before being gavaged with water (control), L. paracasei, or E. coli and maintained on a CD or submitted to a HFD for 8 weeks. (A) Daily food intake per mouse starting from week 3 following L. paracasei or E. coli colonization. (B) Body weight gain. (C and D) Terminal blood serum 1-h fasting levels of (C) leptin and (D) total cholesterol. (E) Terminal liver TG content expressed in micromoles per gram of liver weight. (F) Terminal blood serum 1-h fasting levels of ApoB-48. (G to I) Confocal imaging of LD stained with BODIPY in jejunum sections. (G) Representative confocal images (of 5 per condition) showing intracellular lipid droplets in mice submitted to a HFD. (H and I) Quantitative imaging analysis of intratissular intestinal LD using Imaris software showing (H) LD number and (I) large LD (>4.95 µm^3^) number. (J to M) Intestinal expression levels assessed by RT-qPCR of (J) PPAR targets involved in fatty acid oxidation (K) and in TG and chylomicron biosynthesis and of (L) *Fabp2* and (M) SREBP2 targets involved in cholesterol homeostasis. Results are normalized to the *Actin* gene and expressed as means of fold change relative to the control ± SEM. In panels A to F, H, and I, results are expressed as means ± SEM. *, *P* < 0.05; **, *P* < 0.01; ***, *P* < 0.001 (one-way ANOVA).

In parallel to the groups of mice maintained on a CD, conventional mice colonized with L. paracasei or E. coli were switched to a HFD for 8 weeks to investigate how host response would be affected by each species. Following the switch to a HFD, gut microbiota was enriched in *Enterobacteriaceae* but L. paracasei and E. coli levels were similar to those observed with a CD (Fig. S4). In contrast to control mice and despite their being maintained on similar diets (Fig. 7A), L. paracasei mice resisted excess weight gain (Fig. 7B), and circulating levels of leptin, usually increased in response to a HFD, were accordingly diminished compared to control levels (Fig. 7C). Moreover, TG circulating levels remained similar among the 3 groups of mice (data not shown), but cholesterol circulating levels almost doubled and hepatic TG content tripled in all groups but the L. paracasei mice (Fig. 7D and E). Circulating levels of ApoB-48 in L. paracasei mice were found to be diminished compared to those in control and E. coli mice (Fig. 7F), mirroring circulating cholesterol levels. Given that L. paracasei mice resisted HFD-induced body weight gain, increased lipid clearance from the bloodstream was very unlikely, suggesting that the reduced ApoB-48 circulating levels in L. paracasei mice were due to lower levels of chylomicron secretion.

10.1128/mBio.01493-18.4 FIG S4  L. paracasei and E. coli gut colonization in SPF mice submitted to a HFD following a microbiota-depleting treatment (*n* = 7 to 8 mice per group). Conventional mice were administered a microbiota-depleting antibiotic treatment before being gavaged with water (control), L. paracasei, or E. coli and were switched to a HFD for 8 weeks. Terminal microbiota composition was assessed by conventional culture methods in the feces (A) and ileum (B). Download FIG S4, TIF file, 0.6 MB. Copyright © 2018 Tazi et al. 2018 Tazi et al. This content is distributed under the terms of the Creative Commons Attribution 4.0 International license.

### L. paracasei colonization, in contrast to E. coli colonization, attenuates intestinal response to HFD.

To confirm our hypothesis, we compared levels of intestinal tissue LD and intestinal and liver expression of genes involved in lipid metabolism in the different groups of mice (Fig. 7G to M). Under conditions of maintenance on a HFD, the number of intestinal LD increased by 500% in E. coli mice, whereas the number remained unchanged in L. paracasei mice, and the total number of LD was eventually higher in E. coli mice than in the mice in the other groups (Fig. 7H). In addition, the total number of large (>4.95 µm^3^) LD was augmented in both control and E. coli mice but not in L. paracasei mice, which showed a smaller amount of large LD than the E. coli mice (Fig. 7I).

Although the HFD had almost no impact on hepatic gene expression levels in all groups of mice (data not shown), with a mere 2-fold decrease of *Fasn* mRNA levels, it induced expected compensatory changes in intestinal gene expression levels in control mice, including *Fasn* downregulation reflecting decreased FA *de novo* biosynthesis (Table S5) and overexpression of genes involved in beta-oxidation (Fig. 7J). However, in L. paracasei mice, intestinal expression of PPAR targets involved in beta-oxidation (*Hmgcs2*, *Cpt1a*) and TG biosynthesis (*Dgat2*) was downregulated compared to control mice on a HFD (Fig. 7J and K), suggesting decreased FA intracellular levels; this, along with the results of LD fluorescence imaging, which did not show modifications of intracellular LD in comparison to CD, supports the idea of decreased dietary fat absorption by enterocytes in L. paracasei mice. Also, *Fabp2*, whose expression is negatively correlated to ApoB-48 expression and chylomicron biosynthesis (30), was specifically overexpressed in L. paracasei mice under conditions of maintenance on a HFD (Fig. 7L), which could also account for decreased chylomicron secretion.

10.1128/mBio.01493-18.9 TABLE S5  Small intestine gene expression levels assessed by RT-qPCR in mice colonized with L. paracasei or E. coli under HFD conditions. Download TABLE S5, DOCX file, 0.1 MB. Copyright © 2018 Tazi et al. 2018 Tazi et al. This content is distributed under the terms of the Creative Commons Attribution 4.0 International license.

Similarly to L. paracasei-treated mice, PPAR targets involved in beta-oxidation and TG biosynthesis were downregulated in the intestine of E. coli-treated mice maintained on a HFD (Fig. 7J and K). However, mRNA levels of microsomal triglyceride transfer protein (encoded by the *Mttp* gene) were reduced compared to the levels seen with L. paracasei mice (Fig. 7K). Importantly, MTTP plays critical roles in chylomicron biosynthesis and secretion, and improper lipidation or MTTP deficiency results in ApoB proteasomal degradation and intracellular accumulation of lipids (31). Together with the LD imaging results, these data support the idea of increased lipid storage associated with aberrant dietary fat absorption and/or with a defect, with regard to the intracellular lipid content, in chylomicron synthesis in E. coli mice. The idea of altered adaptation to HFD resulting from increased lipid storage in E. coli mice is further supported by the specific downregulation of SREBP-2 targets compared to CD (Fig. 7M). Indeed, the downregulation of SREBP-2 target genes, solely dependent on intracellular cholesterol content (7), suggested increased intracellular cholesterol levels and/or altered cholesterol trafficking in the small intestine of E. coli mice under conditions of maintenance on a HFD.

## DISCUSSION

The molecular mechanisms and the bacterial effectors involved in microbiota regulation of lipid absorption and metabolism by the intestinal epithelium remain poorly understood. Lactobacilli and E. coli are among the gut’s earliest colonizers, particularly in the small intestine, where bacterial density and diversity steadily increase from the duodenum to the jejunum and then the ileum, where the density approaches that found in the colon (32). The jejunum, which represents the optimal segment for lipid absorption (33), is particularly enriched in *Firmicutes* (especially lactobacilli and streptococci). Moreover, lactobacilli are dominant in the mucus layer and epithelial crypts of the small intestine (34) where proteobacterial species, particularly E. coli, represent the third-most-abundant bacterial phylum (35, 36). L. paracasei and E. coli can hence be considered bona fide model microorganisms suitable for the study of bacterial implications with respect to lipid absorption by enterocytes in the small intestine.

Dietary fat absorption and LD assembly and subsequent storage in cytosolic LD or secretion as chylomicrons and FFA by enterocytes have been extensively studied (23, 33). However, their regulation by environmental factors such as the microbiota remains only partially elucidated (9, 33, 37). In a zebrafish model, the microbiota stimulates FA uptake by enterocytes and induces an enrichment of intestinal LD (9). Monoassociation experiments performed using *Firmicutes* species of the genus *Exiguobacterium*, or *Pseudomonas* proteobacteria, resulted in *Firmicutes*-mediated increases in enterocyte LD numbers associated with robust FA export to the liver and in a non-Firmicutes bacterium-mediated increase in the size of enterocyte LD.

Combining murine and cellular models of enterocyte lipid absorption with representative commensal strains, we showed that under normal diet conditions, the *Firmicutes* species L. paracasei induced a shift toward LD of larger size, whereas the proteobacterium E. coli was associated with reduced LD size and diminished chylomicron circulating levels. These discrepancies reflect important differences between the experimental conditions and the difficulty of defending a universal model of bacterial regulation of host metabolism. Importantly, beyond the relevance of the bacterial species selected, the metabolic differences mediated by the bacteria in interaction with the epithelium that result from the enzymatic modification of nutrients by the bacteria and from the secretion of bacterial soluble factors, notably, lactate and acetate, which represent the main end products of L. paracasei and E. coli fermentation, respectively, and which have been shown to impact cell cycle and metabolism (38), are likely to play a key role in the modulation of lipid absorption and metabolism, as suggested by the *in vitro* reproduction seen in conditioned medium.

**FIG 8:**
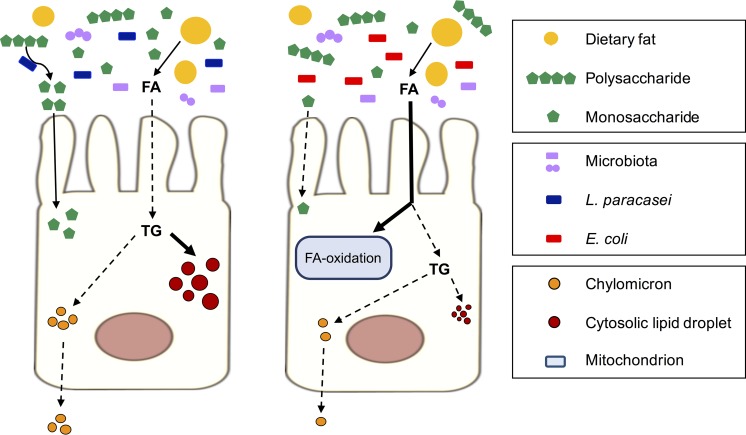
Model for L. paracasei and E. coli impact on lipid absorption and secretion by enterocytes. Once taken up by the brush border of the enterocytes, FA released by dietary lipid micelles are either reesterified in TG or degraded through beta-oxidation. Newly synthetized TG are incorporated into cytosolic LD or into chylomicrons, which are then secreted in the lymphatic system. Under normal chow diet conditions, L. paracasei participates in digestion of polysaccharides, provides monosaccharides to epithelial cells, and decreases FA absorption, and the newly synthetized TG are preferentially stored in cytosolic LD. Conversely, E. coli competes for monosaccharides and disaccharides with epithelial cells and induces an increase in FA absorption and beta-oxidation, leading to reduced TG synthesis, LD size, and chylomicron secretion.

Both L. paracasei and E. coli inhibited lipid secretion *in vitro*, whereas decreased chylomicron circulating levels *in vivo* were observed only under HFD conditions for L. paracasei mice and under CD conditions for E. coli mice. Both bacterial species potentially affected every physiological step leading to chylomicron secretion, i.e., (i) fat absorption, (ii) lipid intracellular metabolism, and (iii) fat incorporation either in prechylomicrons or in the cytoplasmic pool of LD (Fig. 8), and the metabolic and colonization properties of each species likely account for their specific impact on enterocyte lipid metabolism and for the differences between the murine and cellular models. Indeed, the substrate range of E. coli is more limited than that of lactobacilli, which can catalyze the breakdown of polysaccharides into monosaccharides, thus making them available for absorption by host cells (39, 40). In addition, although metabolically active, L. paracasei does not actively multiply in the intestine (41) and competes less for nutrients. Therefore, L. paracasei gut colonization could result in higher levels of energy extraction from complex polysaccharides and, consequently, in lower levels of fat absorption, whereas E. coli colonization might lead to enterocyte deprivation with respect to energy sources from carbohydrates and to increased levels of dietary fat absorption and FA beta-oxidation to sustain mitochondrial metabolism and generation of energy. Future studies will investigate these hypotheses through metabolomics analysis and measurements of energy and lipids in the feces and in the mesenteric lymph following oral fat load treatment of mice.

Conversely, L. paracasei bacteria actively multiply in enterocyte cell culture medium and compete with E. coli for nutrients, which probably accounts for the discrepancies between our cellular and murine models. Besides, these discrepancies reflect the complexity of murine models which encompass diverse microbiota and many regulatory pathways, such as bile acid metabolism pathways, which are absent or presumably silent *in vitro* but participate in the regulation of lipid metabolism *in vivo*.

Nevertheless, L. paracasei and E. coli induced distinct effects on FA intracellular fate both *in vitro* and *in vivo*, i.e., under normal diet conditions, showing increased storage in cytosolic LD reflected by an increased proportion of large LD and enhanced catabolism, respectively (Fig. 8). Therefore, we propose a model in which the modification of the LD size distribution in the presence of L. paracasei is due to a switch from TG integration in chylomicrons to TG storage in cytoplasmic LD. The reesterification of dietary FA in TG by the enterocytes uses two pathways, i.e., the monoacylglycerol pathway and the glycerol 3-phosphate (G3P) pathway (33). Interestingly, the majority of the TG produced by the G3P pathway enters a cytoplasmic pool of LD instead of being incorporated into prechylomicrons. Thus, the hypothesis of predominant activation of this pathway upon L. paracasei stimulation will be investigated in the future.

Although L. paracasei colonization and E. coli colonization did not drastically alter host lipid metabolism under normal diet conditions, they showed dramatically different consequences in animals submitted to a HFD. Because of the relatively low TG content of the diet and the relatively short duration of the experiment compared to other studies (19, 42, 43), HFD maintenance was not accompanied by major modifications of hepatic gene expression or by increased circulating TG and ApoB-48 levels. Nevertheless, L. paracasei colonization buffered HFD-induced alterations of intestinal LD content, chylomicron secretion, liver TG content, and weight gain. The beneficial impact of L. paracasei colonization is presumably linked to decreased dietary fat absorption as discussed above and observed elsewhere (44, 45). Besides, L. paracasei colonization was accompanied by colonization by other lactobacilli which have been shown to directly inhibit dietary fat absorption (43, 44, 46). Conversely, E. coli gut colonization had no beneficial impact on mouse metabolic parameters and dramatically increased intestinal LD content. This altered adaptation, exemplified by the absence of any modification of beta-oxidation gene expression in the intestine of E. coli mice, might have been due to (i) E. coli-mediated increases in energy extraction from fat, which, under HFD conditions, led to excessive fat absorption and probably overcame enterocyte FA degradation and chylomicron synthesis capacities, and to (ii) decreased intestinal amounts of butyrate producer microorganisms subsequent to the dysbiosis induced by E. coli colonization. Indeed, the SCFA butyrate mainly produced by *Clostridia* isolates possesses anti-inflammatory properties and directly stimulates PPAR-γ signaling to enhance beta-oxidation and prevent *Enterobacteriaceae* expansion (47). Moreover, HFD-induced complications have been associated with increased E. coli mucosal adherence and translocation (13). Hence, the substantial E. coli gut colonization levels of our model might potentiate HFD-induced modifications and worsen HFD pathophysiological consequences with respect to host metabolism.

In conclusion, we developed relevant animal and cellular assays to demonstrate that the presence of two commensal bacterial species, L. paracasei and E. coli, had distinct consequences with respect to lipid metabolism and that, under normal diet conditions, those species were associated with increased lipid storage in cytosolic LD and enhanced lipid catabolism, respectively. In addition, L. paracasei gut colonization promoted resistance to HFD-induced alterations through decreased chylomicron secretion. The specific bacterial effectors responsible for these properties will be identified in future studies based on the screening of libraries of tagged mutants, allowing a better understanding of the true molecular processes involved in bacterial regulation of host metabolism.

## MATERIALS AND METHODS

### Animals.

Animals were housed in the Institut Pasteur animal facility. Work on animals was performed in compliance with French and European regulations on care and protection of laboratory animals (EC Directive 2010/2063, French Law 2013-118, 6 February 2013). All experiments were approved by the Ethics Committee and registered under reference 2014-0074.

SPF mice (8-week-old C57BL/6JRj females; Janvier, France) were randomly assigned to 3 experimental groups (*n* = 7 to 8 mice per group) and administered a microbiota-depleting antibiotic treatment, as previously described (22). Briefly, antibiotic treatment started with 3 days of amphotericin B 0.1 mg/ml, administered by gavage daily. From day 3, water flasks were supplemented with ampicillin 1 g/liter and with an antibiotic concoction consisting of vancomycin 5 mg/ml, neomycin 10 mg/ml, metronidazole 10 mg/ml, and amphotericin B 0.1 mg/ml, administered by antibiotic gavage daily for 7 days. A gavage volume of 10 ml/kg of body weight was delivered with a stainless steel tube without prior sedation of the mice. From the day following the end of the microbiota-depleting treatment, mice were administered water (control), E. coli (5.10^8^ CFU, 3 consecutive days), or L. paracasei (5.10^8^ CFU, 3 consecutive days, weekly) by oral gavage.

Immediately after the first bacterial oral gavage, mice of each experiment group were randomly split into 2 groups fed for 8 weeks with either a CD (R03-40; SAFE, France) (13% energy from fat) or HFD (235HF; SAFE, France) (45% energy from fat; cholesterol, 0.17 mg/g), both sterilized by gamma irradiation. The experiment was carried out twice. Mice were weighed weekly, and fresh stool samples were taken up. Mice were killed by cervical dislocation after 1 h of fasting, and blood, liver, and small intestine were recovered and immediately used for measurement of serum parameters (see the supplemental material), histological procedures (jejunum), RNA extraction (proximal ileum), and bacterial counts (distal ileum).

### Cells.

The m-ICcl2 murine intestinal epithelial cell line was cultured as previously described (28) and seeded on Costar transwell plates with a 0.4-µm-pore-size filter (Thermo Fisher) at a density of 3.10^5^/cm^2^. The medium was changed every 2 to 3 days until complete differentiation occurred (14 to 21 days). Bacteria (multiplicity of infection of 100) were added in the upper compartment of the cell chamber, and, following 16 h of incubation, culture supernatants from the upper and lower compartments were collected and cells washed twice in phosphate-buffered saline (PBS) before lysis unless otherwise specified. Cell viability was monitored using the CytoTox 96 cytotoxicity assay (Promega).

### Bacterial strains.

L. paracasei ATCC 334 (formerly referred to as Lactobacillus casei ATCC 334) and a nonpathogenic murine E. coli isolate (13) were grown at 37°C in aerobic atmosphere in MRS medium and TS medium (Thermo Fisher), respectively. Bacteria in stationary-growth phase were harvested by centrifugation, washed with PBS, and resuspended in cell culture medium and water for cellular and animal experiments, respectively. To identify specific effectors, bacteria were incubated in m-ICcl2 medium for 16 h, and bacterial pellets were collected, subjected to heat treatment (110°C, 30 min), and resuspended. Culture supernatants (CS) were filtered (pore size, 0.22 µm). Medium supernatants resulting from 16-h cell-bacterium cocultures were filtered (pore size, 0.22 µm) and used as conditioned medium (CM).

### Bacteriologic analyses.

Stool samples and ileum were homogenized in 2-ml tubes containing a mixture of 1.4-mm-diameter and 2.8-mm-diameter glass beads and 1 ml sterile water using a Precellys system (Bertin Technologies). Serial dilutions were plated on selective media (see the supplemental material) for bacterial identification by matrix-assisted laser desorption ionization–time of flight (MALDI-TOF) mass spectrometry (Bruker) and CFU counts.

### Histological procedures and staining.

Jejunum was cut in pieces (1 cm long) and embedded in OCT compound prior to frozen sectioning on a microtome-cryostat. Staining was done on 7-µm sections. Actin was labeled with Alexa Fluor 647 phalloidin (Thermo Fisher) (1:40), neutral lipids with BODIPY 505/515 (Thermo Fisher) (1:200; stock solution, 1 g/liter), and nuclei with DAPI (4′,6-diamidino-2-phenylindole; Thermo Fisher) (1:5,000).

### RNA isolation and quantitative real-time PCR (RT-qPCR).

Distal jejunum and liver left lobe were homogenized in 2-ml and 7-ml tubes containing a mix of 1.4-diameter and 2.8-mm-diameter glass beads and 1 ml and 2 ml Trizol, respectively, using a Precellys system (Bertin Technologies). RNA extractions from organs and cultured cells were performed using a NucleoSpin RNA II kit (Macherey-Nagel) before cDNA synthesis and RT-qPCR were performed with the primers listed in Table S6 in the supplemental material.

10.1128/mBio.01493-18.10 TABLE S6  Primers used for RT-qPCR (5′–3′). Download TABLE S6, DOCX file, 0.1 MB. Copyright © 2018 Tazi et al. 2018 Tazi et al. This content is distributed under the terms of the Creative Commons Attribution 4.0 International license.

### Western blotting.

Cells were lysed in 300 µl of radioimmunoprecipitation assay (RIPA) buffer, and the lysate was heated in Laemmli buffer for 5 min at 90°C before migration in acrylamide SDS-PAGE. Proteins were transferred onto polyvinylidene difluoride (PVDF) membranes, incubated with the primary antibodies (see the supplemental material) overnight at 4°C in 5% bovine serum albumin (BSA)–PBS, washed in PBS–Tween 0.1%, incubated with a peroxidase-labeled secondary antibody (1:10,000) for 1 h, and revealed by the use of ECL chemiluminescence reagent (Thermo Fisher). Image acquisitions and quantifications were performed with an Amersham Imager 600 system (GE Healthcare). Experiments were performed 3 times in duplicate.

### Reagents.

Triglyceride quantification (Abnova) and Amplex Red cholesterol assay kits used in the experiments were from Thermo Fisher, and a mouse ApoB-48 enzyme-linked immunosorbent assay (ELISA) kit was obtained from Cusabio. Other mouse ELISA kits (including tumor necrosis factor alpha [TNF-α] Quantikine high-sensitivity [HS], Leptin Quantikine, and CXCL1/KC Quantikine kits) were from R&D Systems.

3-Benzyl-5-[(2-nitrophenoxy)methyl]-dihydrofuran-2(3H)-one (3BDO) (Sigma-Aldrich) was added in the cell culture medium at 60 µM together with the bacteria.

### Fluorescent lipid micelle assay.

BODIPY 558/568 C_12_ FA (Thermo Fisher; D-3835) was stored at stock concentrations in chloroform at −20°C. Lipid micelles were prepared as previously described (48) in serum-free medium with a final BODIPY C_12_ FA concentration of 0.02 mM.

After 16 h of cell-bacterium coculture, the cellular medium of the upper compartment was replaced by 500 µl of the lipid micelle preparation (time zero). Following 10 min of incubation at 37°C in a 5% CO_2_ atmosphere, the lipid micelle preparation was replaced by regular serum-free medium. Fluorescence in the lower compartment was measured using an Infinite 200 PRO plate reader (Tecan). Cells were washed twice in PBS before fixation in 4% paraformaldehyde (PFA) was performed for LD measurements.

### Image acquisition and analysis.

Images from jejunum sections (1 per animal) were randomly acquired using an Opterra swept-field confocal microscope (Bruker). Areas of interest (2 per animal) composed of intestinal villi (>4,200 µm^2^) were delineated manually using Fiji software. Images of cultured m-ICcl2 cells were randomly acquired using confocal microscopy (Leica SP5).

Intestinal and cellular LD were quantified using BODIPY fluorescence with Imaris software (Bitplane). For each image, we applied a segmentation protocol to identify individual objects (LD) and to measure their volume.

### Statistical analysis.

Data were analyzed using GraphPad Prism 5.0 software. Continuous variables were compared using one-way analysis of variance (ANOVA). A *P* value of ≤0.05 was considered significant.
