# Supplementary figures and images for: Disentangling Host-Microbiota Regulation of Lipid Secretion by Enterocytes: Insights from Commensals Lactobacillus paracasei and Escherichia coli
Source: mBio. 2018 Sep 4;9(5):e01493-18. doi: 10.1128/mBio.01493-18 (PMC6123438; doi:10.1128/mBio.01493-18)

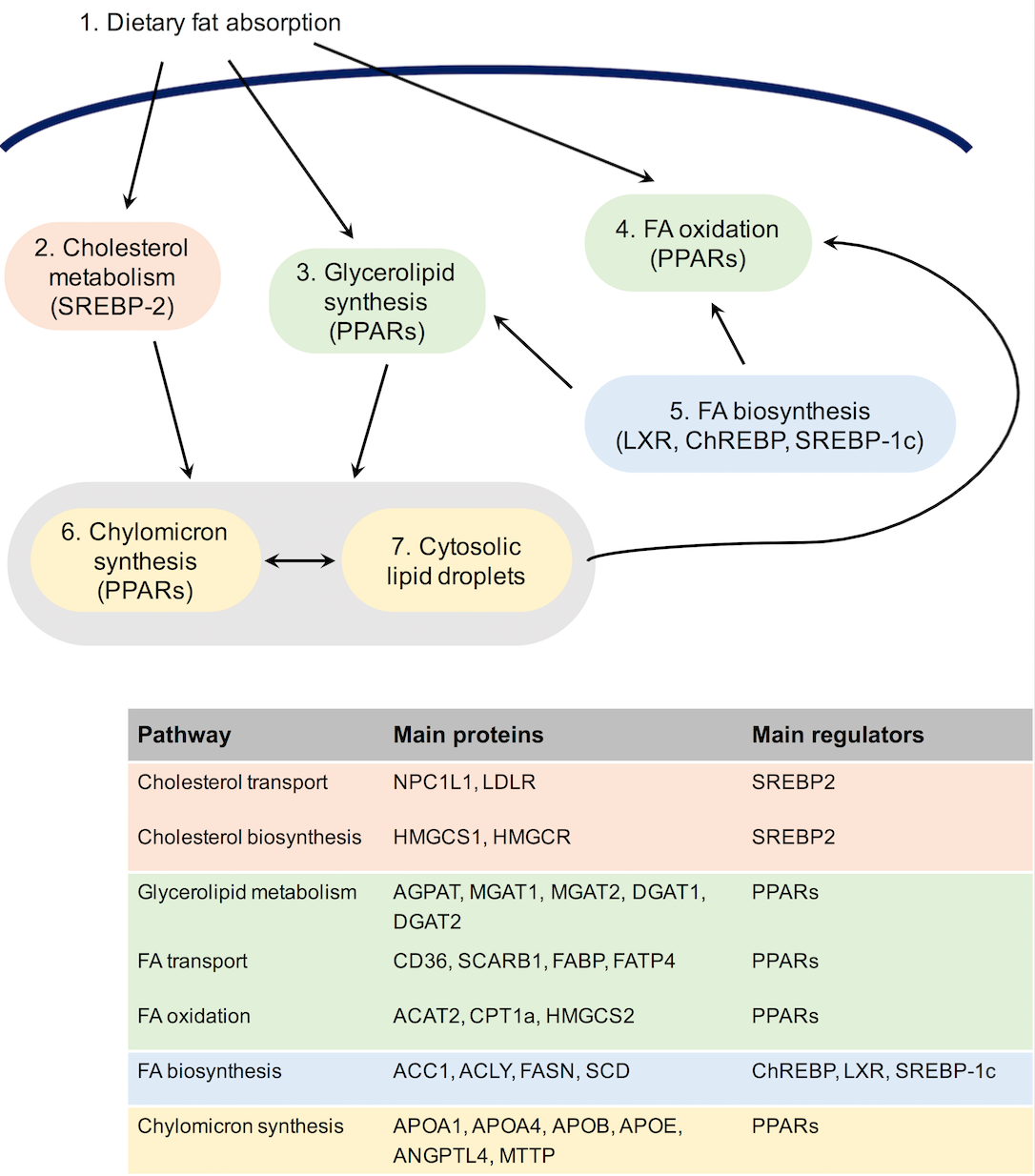

Supplement: FIG S1 [file mbo004184048sf1.tif]

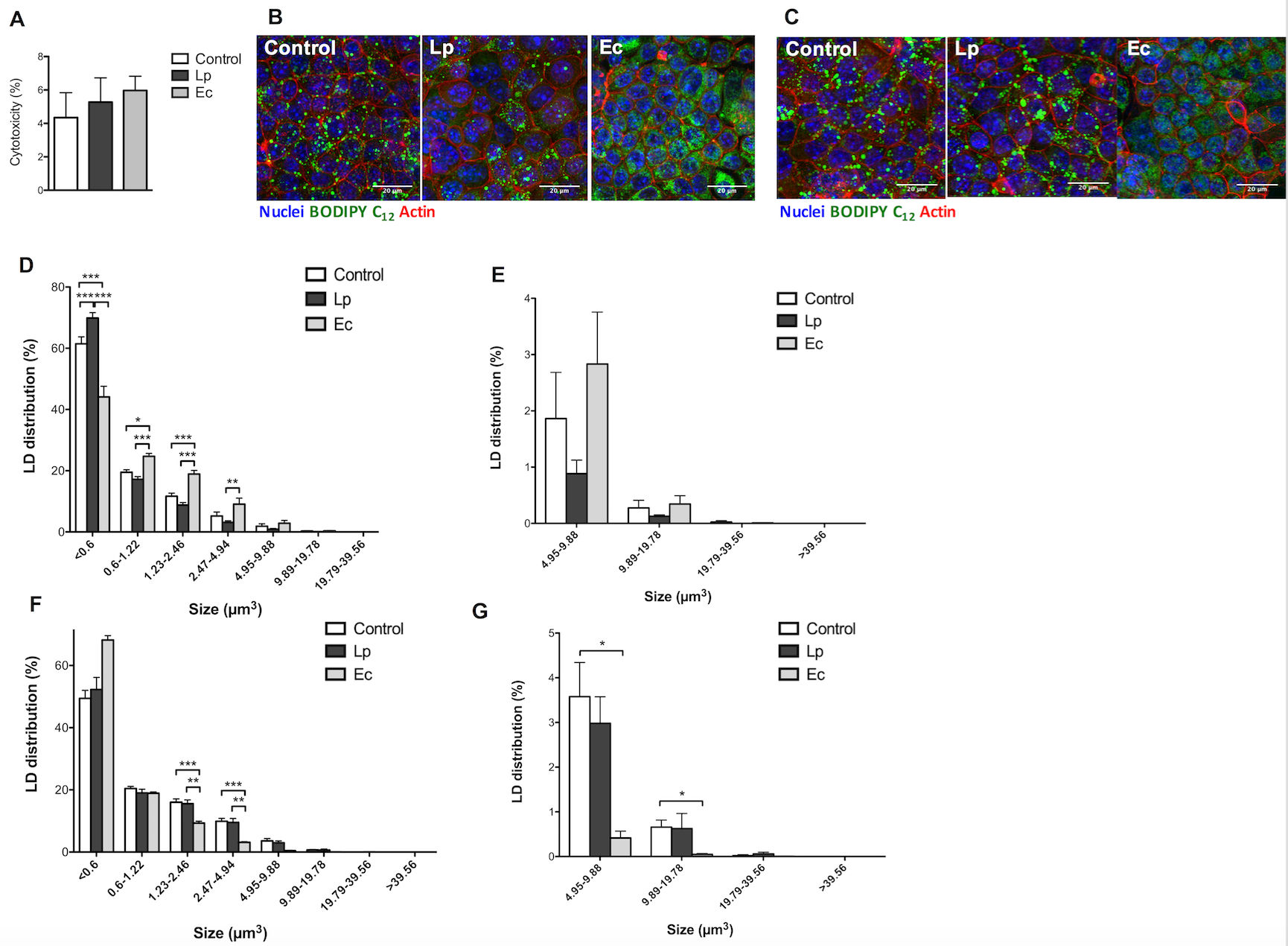

Supplement: FIG S2 [file mbo004184048sf2.tif]

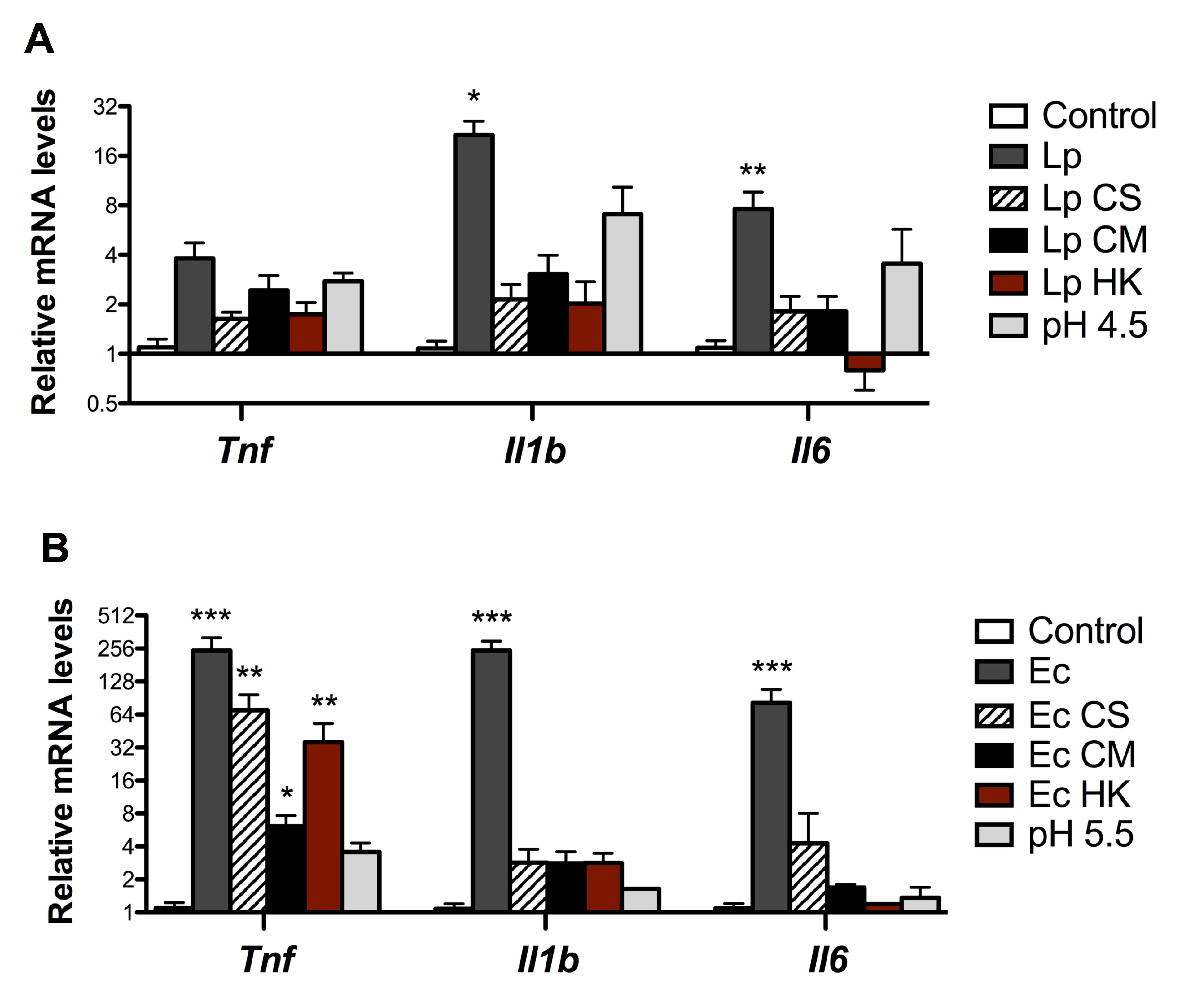

Supplement: FIG S3 [file mbo004184048sf3.tif]

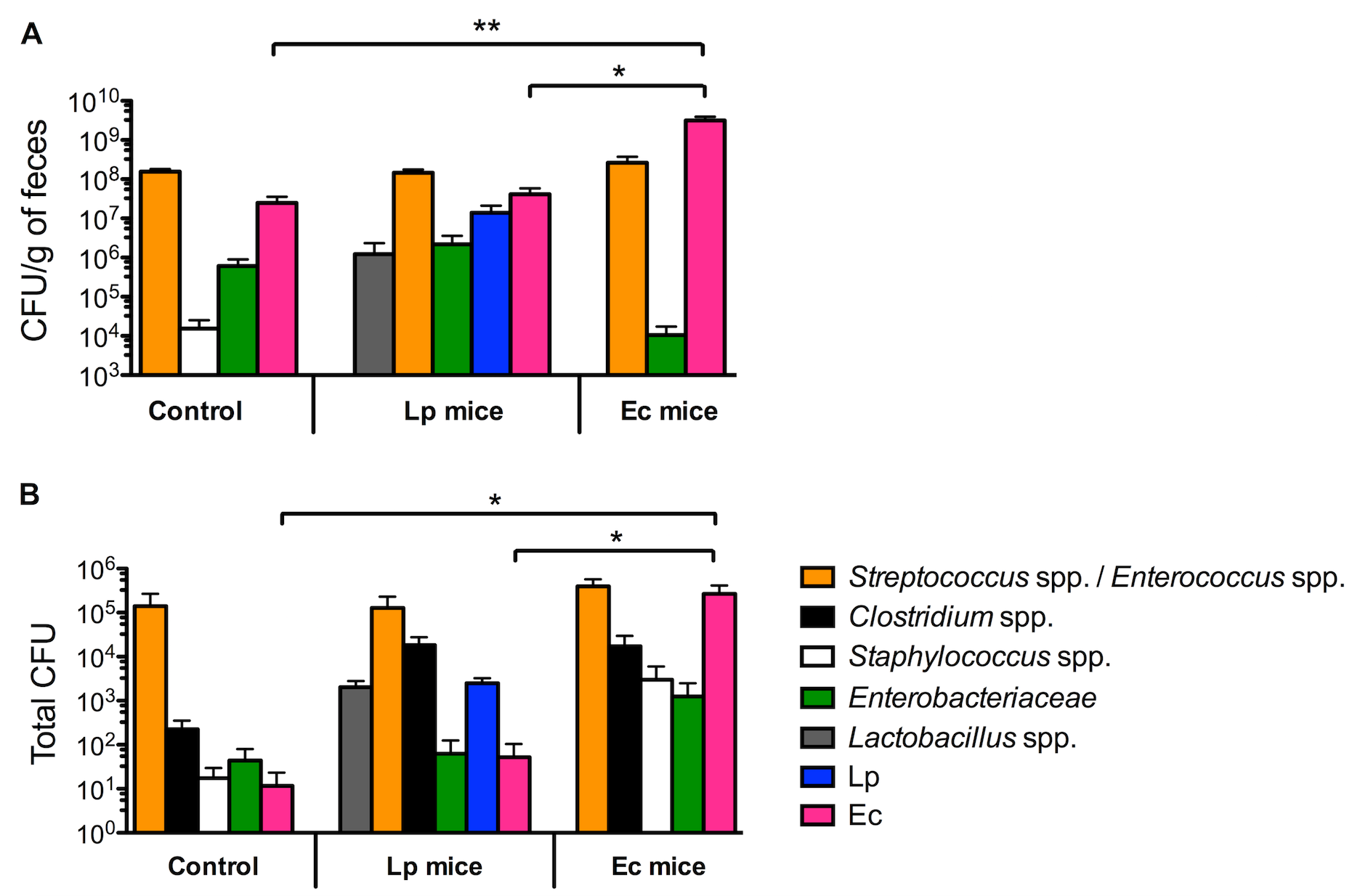

Supplement: FIG S4 [file mbo004184048sf4.tif]
