# Supplementary material for: Disentangling Host-Microbiota Regulation of Lipid Secretion by Enterocytes: Insights from Commensals Lactobacillus paracasei and Escherichia coli
Source: mBio. 2018 Sep 4;9(5):e01493-18. doi: 10.1128/mBio.01493-18 (PMC6123438; doi:10.1128/mBio.01493-18)
Supplement: TABLE S2 [file mbo004184048st2.docx]

**Table S2. Hepatic gene expression levels assessed by RT-qPCR in mice colonized with Lp or Ec in chow diet.**

|  |  |  | **Lp** | | **Ec** | | |
| --- | --- | --- | --- | --- | --- | --- | --- |
| **Gene symbol** | **Gene name (main alias)** | **Main related pathway/function** | **Fold change^a^ (mean ± SEM)** | ***P*^b^** | **Fold change^a^ (mean ± SEM)** | ***P*^b^** | ***P*_Lp_^c^** |
| *Acaca* | Acetyl-CoA carboxylase alpha (*Acc1*) | Fatty acid biosynthesis | 0.73 ± 0.20 | NS | 1.01 ± 0.07 | NS | NS |
| *Acat1*^d^ | Acetyl-CoA Acetyltransferase 1 | Synthesis of ketone bodies | 5.13 ± 1.60 | <0.01 | 1.88 ± 0.37 | NS | <0.05 |
| *Acat2*^d^ | Acetyl-CoA Acetyltransferase 2 | Fatty acid degradation | 3.59 ± 0.96 | <0.05 | 1.66 ± 0.68 | NS | NS |
| *Acly* | ATP Citrate Lyase | Acetyl-CoA biosynthesis | 2.84 ± 0.92 | NS | 4.50 ± 0.71 | <0.05 | NS |
| *Angptl4*^d^ | Angiopoietin Like 4 (*Fiaf*) | Lipoprotein metabolism | 1.42 ± 0.38 | NS | 1.28 ± 0.24 | NS | NS |
| *Apoa1*^d^ | Apolipoprotein A1 | Lipoprotein metabolism | 2.76 ± 0.93 | NS | 1.02 ± 0.78 | NS | NS |
| *Apob*^d^ | Apolipoprotein B | Lipoprotein metabolism | 3.54 ± 0.93 | <0.01 | 1.33 ± 0.27 | NS | <0.05 |
| *Apoe*^d^ | Apolipoprotein E | Lipoprotein metabolism | 5.24 ± 1.23 | <0.05 | 1.69 ± 0.25 | NS | NS |
| *Ccl2* | C-C Motif Chemokine Ligand 2 (*Mcp-1*) | Chemokine | 0.58 ± 0.16 | NS | 0.45 ± 0.16 | NS | NS |
| *Ccl3* | C-C Motif Chemokine Ligand 3 (*Mip-1a*) | Chemokine | 0.65 ± 0.14 | NS | 0.92 ± 0.20 | NS | NS |
| *Ccl5* | C-C Motif Chemokine Ligand 5 (*Rantes*) | Chemokine | 1.78 ± 0.49 | NS | 1.31 ± 0.20 | NS | NS |
| *Cd36*^d^ | CD36 Molecule (*Scarb3*) | Fat absorption | 2.41 ± 0.79 | NS | 1.32 ± 0.35 | NS | NS |
| *Chrebp* | Carbohydrate responsive element binding protein | Lipogenesis/ Transcriptional regulator | 2.72 ± 0.44 | <0.01 | 1.65 ± 0.09 | NS | NS |
| *Cpt1a*^d^ | Carnitine Palmitoyltransferase 1A | Fatty acid degradation | 5.56 ± 1.44 | <0.01 | 2.37 ± 0.53 | NS | <0.05 |
| *Dgat1*^d^ | Diacylglycerol O-Acyltransferase 1 | TG biosynthesis | 1.66 ± 0.33 | NS | 1.19 ± 0.22 | NS | NS |
| *Dgat2*^d^ | Diacylglycerol O-Acyltransferase 1 | TG biosynthesis | 5.86 ± 1.49 | <0.01 | 2.26 ± 0.50 | NS | NS |
| *Fabp1*^d^ | Fatty Acid Binding Protein 1 (liver) | Fatty acid transport | 0.72 ± 0.15 | NS | 0.85 ± 0.16 | NS | NS |
| *Fasn* | Fatty Acid Synthase | Fatty acid biosynthesis | 0.68 ± 0.06 | NS | 0.68 ± 0.12 | NS | NS |
| *Fatp4*^d^ | Fatty Acid Transport Protein 4 | Fatty acid transport | 3.58 ± 1.68 | NS | 1.23 ± 0.17 | NS | NS |
| *Hmgcr* | 3-Hydroxy-3-Methylglutaryl-CoA Reductase | Cholesterol biosynthesis | 4.49 ± 1.29 | <0.01 | 1.69 ± 0.37 | NS | <0.01 |
| *Hmgcs1* | 3-Hydroxy-3-Methylglutaryl-CoA Synthase 1 | Cholesterol biosynthesis | 3.89 ± 1.51 | <0.05 | 1.44 ± 0.43 | NS | <0.05 |
| *Hmgcs2* | 3-Hydroxy-3-Methylglutaryl-CoA Synthase 1 | Ketogenesis | 3.52 ± 0.66 | <0.05 | 1.20 ± 0.15 | NS | NS |
| *Il1a* | Interleukin 1 Alpha | Cytokine | 5.78 ± 1.64 | NS | 8.51 ± 0.50 | <0.05 | NS |
| *Il1b* | Interleukin 1 Beta | Cytokine | 0.56 ± 0.09 | <0.05 | 0.60 ± 0.09 | <0.05 | NS |
| *Il6* | Interleukin 6 | Cytokine | 1.00 ± 0.26 | NS | 1.07 ± 0.15 | NS | NS |
| *Ldlr* | Low Density Lipoprotein Receptor | Lipoprotein metabolism | 6.67 ± 1.80 | <0.01 | 1.85 ± 0.60 | NS | <0.01 |
| *Lxra* | Liver X Receptor Alpha | Lipoprotein metabolism/ Nuclear receptor | 1.64 ± 0.31 | NS | 1.01 ± 0.16 | NS | NS |
| *Mttp*^d^ | Microsomal Triglyceride Transfer Protein | Lipoprootein metabolism | 1.00 ± 0.22 | NS | 0.90 ± 0.12 | NS | NS |
| *Ppara*^d^ | Peroxisome Proliferator Activated Receptor Alpha | Transcriptional regulator | 0.96 ± 0.15 | NS | 0.79 ± 0.07 | NS | NS |
| *Ppard*^d^ | Peroxisome Proliferator Activated Receptor Beta/Delta | Transcriptional regulator | 1.03 ± 0.37 | NS | 0.88 ± 0.10 | NS | NS |
| *Pparg*^d^ | Peroxisome Proliferator Activated Receptor Gamma | Transcriptional regulator | 10.60 ± 3.28 | <0.01 | 2.32 ± 0.56 | NS | <0.01 |
| *Scarb1* | Scavenger Receptor Class B Member 1 | Lipoprotein metabolism/ Fat absorption | 1.86 ± 0.28 | NS | 1.24 ± 0.18 | NS | NS |
| *Scd*^d^ | Stearoyl-CoA Desaturase | Fatty acid biosynthesis | 3.69 ± 0.78 | <0.01 | 1.55 ± 0.15 | NS | <0.01 |
| *Srebf1* | Sterol Regulatory Element Binding Transcription Factor 1 | Lipogenesis/ Transcriptional regulator | 4.69 ± 0.76 | <0.01 | 1.94 ± 0.50 | NS | NS |
| *Srebf2* | Sterol Regulatory Element Binding Transcription Factor 2 | Lipogenesis/ Transcriptional regulator | 4.50 ± 1.66 | <0.01 | 1.80 ± 0.35 | NS | NS |
| *Tnf* | Tumor Necrosis Factor | Cytokine | 3.19 ± 1.20 | NS | 1.51 ± 0.47 | NS | NS |

^a^Conventional mice (n=7-8 per group) were administered a microbiota depleting antibiotic treatment before being gavaged with water (control), Lp or Ec and maintained in chow diet for 8 weeks. Results are normalized to *Actin* and expressed as mean fold change relative to control +/- SEM.

^b^Statistical significance compared to control mice.

^c^Statistical significance compared to Lp colonized mice.

^d^PPAR pathway controlled genes.

NS : not significant.
