# Supplementary material for: Disentangling Host-Microbiota Regulation of Lipid Secretion by Enterocytes: Insights from Commensals Lactobacillus paracasei and Escherichia coli
Source: mBio. 2018 Sep 4;9(5):e01493-18. doi: 10.1128/mBio.01493-18 (PMC6123438; doi:10.1128/mBio.01493-18)
Supplement: TABLE S4 [file mbo004184048st4.docx]

**Table S4. Impact of bacterial factors and acidic pH on m-ICcl2 enterocytes^a^.**

|  | **Lp** |  |  | | | | |  | **Ec** | | | | |
| --- | --- | --- | --- | --- | --- | --- | --- | --- | --- | --- | --- | --- | --- |
|  | **Live** | **CS** | **CM** | **HK** | **pH 4.5** | **CS + HK** | **CM + HK** |  | **Live** | **CS** | **CM** | **HK** | **pH 5.5** |
| **Fluoresccent lipid secretion, 4h (% of control)^b^** | | | | | |  |  | | | | |  |  |
|  | 60 ± 4 | 92 ± 10 | 105 ± 10 | 105 ± 13 | 120 ± 5 | 41 ± 3 | 42 ± 4 |  | 42 ± 6 | 79 ± 5 | 65 ± 15 | 107 ± 12 | 92 ± 5 |
| **Lipid concentrations (% of control)^c^** | | | | | |  | | | | | |  |  |
| TG, Intracellular | 134 ± 5 | 111 ± 13 | 97 ± 4 | 95 ± 6 | 81 ± 5 | 123 ± 6 | 125 ± 4 |  | 56 ± 13 | 91 ± 12 | 89 ± 7 | 102 ± 6 | 115 ± 18 |
| TG, UC | 31 ± 5 | 100 ± 4 | 107 ± 11 | ND | 77 ± 7 | 73 ± 5 | 82 ± 9 |  | 17 ± 3 | 111 ± 8 | 93 ± 8 | ND | 94 ± 2 |
| TG, LC | 66 ± 2 | 104 ± 5 | 121 ± 6 | ND | 94 ± 5 | 99 ± 3 | 95 ± 6 |  | 65 ± 2 | 95 ± 3 | 94 ± 4 | ND | 92 ± 4 |
| **mRNA levels (fold change)^d^** | | | | | |  | | | | | |  |  |
| *Srebf1* | 0.43 ± 0.04 | 0.74 ± 0.05 | 0.53 ± 0.10 | 1.02 ± 0.05 | 0.84 ± 0.14 | ND | ND |  | 0.51 ± 0.08 | 0.66 ± 0.03 | 0.66 ± 0.04 | 0.85 ± 0.13 | 0.86 ± 0.08 |
| *Acaca* | 0.47 ± 0.07 | 0.93 ± 0.08 | 0.72 ± 0.02 | 1.37 ± 0.07 | 1.31 ± 0.43 | ND | ND |  | 0.51 ± 0.11 | 0.76 ± 0.05 | 0.98 ± 0.11 | 1.24 ± 0.21 | 1.10 ± 0.39 |
| *Fasn* | 0.40 ± 0.06 | 0.94 ± 0.05 | 0.73 ± 0.04 | 1.55 ± 0.16 | 1.08 ± 0.23 | ND | ND |  | 0.73 ± 0.13 | ND | ND | ND | ND |
| *Fabp2* | 7.19 ± 1.30 | 3.56 ± 0.60 | 3.58 ± 0.81 | 2.23 ± 0.55 | 4.11 ± 1.67 | ND | ND |  | 11.19 ± 3.29 | 1.14 ± 0.05 | 1.48 ± 0.13 | 2.24 ± 0.40 | 3.45 ± 1.45 |
| *Hmgcs2* | 7.18 ± 1.06 | 2.43 ± 0.33 | 3.46 ± 0.63 | 3.04 ± 0.37 | 3.53 ± 1.01 | ND | ND |  | 12.51 ± 2.93 | 2.31 ± 0.38 | 2.47 ± 0.24 | 1.60 ± 0.89 | 1.90 ± 0.87 |
| *Acat2* | 1.22 ± 0.36 | ND | ND | ND | ND | ND | ND |  | 5.43 ± 1.09 | 3.83 ± 0.17 | 3.74 ± 0.16 | 1.22 ± 0.18 | 0.71 ± 0.12 |
| *Mttp* | 1.12 ± 0.21 | ND | ND | ND | ND | ND | ND |  | 3.52 ± 0.71 | 1.38 ± 0.22 | 1.02 ± 0.22 | 0.83 ± 0.18 | 4.25 ± 1.37 |
| *Apoa4* | 1.64 ± 0.24 | ND | ND | ND | ND | ND | ND |  | 2.18 ± 0.42 | 0.68 ± 0.13 | 0.69 ± 0.06 | 0.98 ± 0.17 | 1.61 ± 0.20 |
| *Apob* | 1.55 ± 0.37 | ND | ND | ND | ND | ND | ND |  | 3.46 ± 0.88 | 0.87 ± 0.35 | 0.54 ± 0.08 | 0.60 ± 0.15 | 1.03 ± 0.09 |

^a^Results are expressed as mean +/- SEM. Boxes in green and orange indicate significant decrease and increase compared to control, respectively (p<0.05, one-way ANOVA).

^b^Fluorescent lipid secretion 4h after stimulation by fluoresccent lipid micelles preceeded by 16h exposition to Lp or Ec-related conditions.

^c^Lipid concentrations following 16h exposition to Lp or Ec-related conditions.

^d^mRNA levels following 16h exposition to Lp or Ec-related conditions by RT-qPCR using *Actin* as reporter gene.

CS : bacterial culture supernatant ; CM : supernatant of cells co-cultured with bacteria ; HK : heat-killed bacteria ; ND : not determined.

Control : non-exposed cells ; Live : live bacteria, MOI 100 ; CS : culture supernatant ; CM : conditioned medium ; HK : heat-killed bacteria ; UC : upper compartment ; LC : lower compartment ; ND : not determined.
