# Supplementary material for: Disentangling Host-Microbiota Regulation of Lipid Secretion by Enterocytes: Insights from Commensals Lactobacillus paracasei and Escherichia coli
Source: mBio. 2018 Sep 4;9(5):e01493-18. doi: 10.1128/mBio.01493-18 (PMC6123438; doi:10.1128/mBio.01493-18)
Supplement: TABLE S5 [file mbo004184048st5.docx]

**Table S5. Small intestine gene expression levels assessed by RT-qPCR in mice colonized with Lp or Ec under HFD.**

|  |  |  | **Control** | | **Lp** | | **Ec** | | |
| --- | --- | --- | --- | --- | --- | --- | --- | --- | --- |
| **Gene symbol** | **Gene name (main alias)** | **Main related pathway/function** | **Fold change^a^ (mean ± SEM)** | ***P*_CD_^b^** | **Fold change^a^ (mean ± SEM)** | ***P*_HFD_^c^** | **Fold change^a^ (mean ± SEM)** | ***P*_HFD_^c^** | ***P*_Lp_^d^** |
| *Acaca* | Acetyl-CoA carboxylase alpha (*Acc1*) | Fatty acid biosynthesis | 0.68 ± 0.11 | NS | 0.79 ± 0.10 | NS | 0.39 ± 0.04 | NS | NS |
| *Acat2*^e^ | Acetyl-CoA Acetyltransferase 2 | Fatty acid degradation | 0.68 ± 0.11 | NS | 1.17 ± 0.23 | NS | 0.55 ± 0.11 | NS | NS |
| *Acly* | ATP Citrate Lyase | Acetyl-CoA biosynthesis | 0.62 ± 0.11 | NS | 1.03 ± 0.21 | NS | 0.60 ± 0.06 | NS | NS |
| *Angptl4*^e^ | Angiopoietin Like 4 (*Fiaf*) | Lipoprotein metabolism | 0.80 ± 0.08 | NS | 0.50 ± 0.09 | NS | 0.42 ± 0.06 | NS | NS |
| *Apoa1*^e^ | Apolipoprotein A1 | Lipoprotein metabolism | 1.06 ± 0.13 | NS | 1.03 ± 0.18 | NS | 0.59 ± 0.08 | NS | NS |
| *Apoa4*^e^ | Apolipoprotein A4 | Lipoprotein metabolism | 4.19 ± 1.30 | <0.05 | 6.18 ± 1.53 | NS | 6.23 ± 1.42 | NS | NS |
| *Apob*^e^ | Apolipoprotein B | Lipoprotein metabolism | 1.02 ± 0.13 | NS | 0.80 ± 0.10 | NS | 0.50 ± 0.08 | <0.01 | NS |
| *Apoe*^e^ | Apolipoprotein E | Lipoprotein metabolism | 1.30 ± 0.25 | NS | 1.03 ± 0.15 | NS | 0.68 ± 0.10 | NS | NS |
| *Ccl2* | C-C Motif Chemokine Ligand 2 (*Mcp-1*) | Chemokine | 0.86 ± 0.28 | NS | 0.99 ± 0.19 | NS | 0.49 ± 0.11 | NS | NS |
| *Ccl3* | C-C Motif Chemokine Ligand 3 (*Mip-1a*) | Chemokine | 3.97 ± 1.35 | <0.05 | 2.07 ± 0.35 | NS | 1.23 ± 0.33 | <0.05 | NS |
| *Ccl5* | C-C Motif Chemokine Ligand 5 (*Rantes*) | Chemokine | 1.10 ± 0.24 | NS | 0.82 ± 0.09 | NS | 0.44 ± 0.06 | NS | NS |
| *Cd36*^e^ | CD36 Molecule (*Scarb3*) | Fat absorption | 1.04 ± 0.16 | NS | 1.23 ± 0.10 | NS | 1.28 ± 0.04 | NS | NS |
| *Chrebp* | Carbohydrate responsive element binding protein | Lipogenesis/ Transcriptional regulator | 1.31 ± 0.16 | NS | 1.47 ± 0.16 | NS | 1.14 ± 0.15 | NS | NS |
| *Cpt1a*^e^ | Carnitine Palmitoyltransferase 1A | Fatty acid degradation | 1.69 ± 0.09 | <.001 | 0.89 ± 0.09 | <0.001 | 0.63 ± 0.06 | <0.001 | NS |
| *Dgat1*^e^ | Diacylglycerol O-Acyltransferase 1 | TG biosynthesis | 1.57 ± 0.42 | NS | 1.65 ± 0.50 | NS | 0.92 ± 0.15 | NS | NS |
| *Dgat2*^e^ | Diacylglycerol O-Acyltransferase 1 | TG biosynthesis | 1.80 ± 0.12 | <0.01 | 0.81 ± 0.11 | <0.001 | 0.66 ± 0.05 | <.001 | NS |
| *Fabp2*^e^ | Fatty Acid Binding Protein 2 (intestinal) | Fatty acid transport | 1.33 ± 0.18 | NS | 2.79 ± 0.49 | <0.01 | 1.15 ± 0.20 | NS | <0.01 |
| *Fasn* | Fatty Acid Synthase | Fatty acid biosynthesis | 0.52 ± 0.08 | <0.01 | 0.60 ± 0.12 | NS | 0.33 ± 0.07 | NS | NS |
| *Fatp4*^e^ | Fatty Acid Transport Protein 4 | Fatty acid transport | 0.93 ± 0.11 | NS | 0.68 ± 0.12 | NS | 0.51 ± 0.08 | NS | NS |
| *Hmgcr* | 3-Hydroxy-3-Methylglutaryl-CoA Reductase | Cholesterol biosynthesis | 0.93 ± 0.14 | NS | 1.16 ± 0.18 | NS | 0.86 ± 0.19 | NS | NS |
| *Hmgcs1* | 3-Hydroxy-3-Methylglutaryl-CoA Synthase 1 | Cholesterol biosynthesis | 1.46 ± 0.26 | NS | 1.53 ± 0.17 | NS | 0.88 ± 0.17 | NS | NS |
| *Hmgcs2*^e^ | 3-Hydroxy-3-Methylglutaryl-CoA Synthase 1 | Ketogenesis | 3.36 ± 0.41 | <0.001 | 0.77 ± 0.19 | <0.001 | 0.61 ± 0.16 | <0.001 | NS |
| *Il1a* | Interleukin 1 Alpha | Cytokine | 1.07 ± 0.22 | NS | 1.01 ± 0.19 | NS | 0.53 ± 0.11 | NS | NS |
| *Il1b* | Interleukin 1 Beta | Cytokine | 1.82 ± 0.42 | NS | 1.48 ± 0.31 | NS | 1.08 ± 0.15 | NS | NS |
| *Il6* | Interleukin 6 | Cytokine | 9.49 ± 4.74 | <0.05 | 3.12 ± 0.64 | NS | 2.01 ± 0.51 | NS | NS |
| *Ldlr* | Low Density Lipoprotein Receptor | Lipoprotein metabolism | 0.88 ± 0.16 | NS | 1.29 ± 0.17 | NS | 0.62 ± 0.11 | NS | NS |
| *Lxra* | Liver X Receptor Alpha | Lipoprotein metabolism/ Nuclear receptor | 2.25 ± 0.50 | NS | 2.19 ± 0.29 | NS | 3.10 ± 0.83 | NS | NS |
| *Mttp*^e^ | Microsomal Triglyceride Transfer Protein | Lipoprotein metabolism | 1.25 ± 0.17 | NS | 1.61 ± 0.17 | NS | 0.74 ± 0.11 | NS | <0.05 |
| *Npc1l1* | Niemann-Pick C1-Like Protein 1 | Intracellular Cholesterol Transporter | 0.63 ± 0.07 | <0.05 | 0.40 ± 0.07 | NS | 0.40 ± 0.08 | NS | NS |
| *Ppara*^e^ | Peroxisome Proliferator Activated Receptor Alpha | Transcriptional regulator | 0.93 ± 0.16 | NS | 1.03 ± 0.22 | NS | 1.04 ± 0.18 | NS | NS |
| *Ppard*^e^ | Peroxisome Proliferator Activated Receptor Beta/Delta | Transcriptional regulator | 1.98 ± 0.22 | <0.01 | 1.45 ± 0.18 | NS | 0.88 ± 0.14 | <0.01 | NS |
| *Pparg*^e^ | Peroxisome Proliferator Activated Receptor Gamma | Transcriptional regulator | 1.39 ± 0.11 | NS | 1.16 ± 0.17 | NS | 0.74 ± 0.04 | NS | NS |
| *Scarb1* | Scavenger Receptor Class B Member 1 | Lipoprotein metabolism/ Fat absorption | 1.07 ± 0.10 | NS | 1.08 ± 0.17 | NS | 0.67 ± 0.11 | NS | NS |
| *Scd*^e^ | Stearoyl-CoA Desaturase | Fatty acid biosynthesis | 0.90 ± 0.32 | NS | 0.86 ± 0.16 | NS | 1.16 ± 0.31 | NS | NS |
| *Srebf1* | Sterol Regulatory Element Binding Transcription Factor 1 | Lipogenesis/ Transcriptional regulator | 2.04 ± 0.37 | NS | 1.71 ± 0.21 | NS | 2.40 ± 0.82 | NS | NS |
| *Srebf2* | Sterol Regulatory Element Binding Transcription Factor 2 | Lipogenesis/ Transcriptional regulator | 1.21 ± 0.15 | NS | 0.89 ± 0.05 | NS | 0.48 ± 0.07 | <0.01 | NS |
| *Tnf* | Tumor Necrosis Factor | Cytokine | 0.54 ± 0.08 | NS | 0.76 ± 0.12 | NS | 0.51 ± 0.15 | NS | NS |

^a^Conventional mice (n=7-8 per group) were administered a microbiota depleting antibiotic treatment before being gavaged with water (control), Lp or Ec and switched to high fat diet (HFD) for 8 weeks. Results are normalized to *Actin* and expressed as mean fold change relative to control maintained in normal chow diet (CD) +/- SEM.

^b^Statistical significance compared to control mice in CD.

^c^Statistical significance compared to control mice in HFD.

^d^Statistical significance compared to Lp colonized mice in HFD.

^e^PPAR pathway controlled genes.

NS : not significant.
