# Supplementary material for: Disentangling Host-Microbiota Regulation of Lipid Secretion by Enterocytes: Insights from Commensals Lactobacillus paracasei and Escherichia coli
Source: mBio. 2018 Sep 4;9(5):e01493-18. doi: 10.1128/mBio.01493-18 (PMC6123438; doi:10.1128/mBio.01493-18)
Supplement: TABLE S6 [file mbo004184048st6.docx]

**Supplemental Material**

**Bacteriological analyses**

Serial dilutions of homogenized stool samples and ileum were plated on 4 media: Columbia agar with 5% horse blood (COH, bioMérieux), Columbia CAP agar with 5% sheep blood (Oxoid) for anaerobes and Gram positive bacteria, lactobacilli MRS agar (ThermoFisher), and UriSelect^TM^ 4 chromogenic medium (Bio-Rad) for non-fastidious bacteria. Plates were incubated 48h at 37°C in aerobic (COH, MRS, UriSelect^TM^ 4) or anaerobic (COH, CAP). Bacteria were identified by mass spectrometry MALDI-TOF Matrix Assisted Laser Desorption Ionisation Time Of Flight) (BrukerTM).

**RNA isolation and quantitative real-time PCR**

cDNA synthesis was performed from 1 μg of RNA using oligo-dT (ThermoFisher) and SuperScript III RT (ThermoFisher). RT-qPCR was performed using Power SYBR Green PCR system (ThermoFisher) and specific primers shown below (1–17) on the QuantStudio 7 Flex Real-Time PCR System (ThermoFisher). Relative quantification of gene expression normalized with *Actin* was performed using the comparative 2^-ΔΔCt^ method.

Table: Primers used for RT-qPCR

| Target | Sequence | Supplemental references |
| --- | --- | --- |
| *Acaca* | F : ggacaccagttttgcattga  R :agtttgggaggacatcgaaa | 1 |
| *Acat1* | F : AGCCTACCGCCTAGACCGCC  R : CCTCCGCCTCTGCTGCCAAC | 2 |
| *Acat2* | F : GACTTGGTGCAATGGACTCG  R : GGTCTTGCTTGTAGAATCTGG | 3 |
| *Acly* | F : ttggatgccgcagcaaagatgttc  R : ttgaggatctgcactcgcatgtct | This study |
| *Actb* | F : gggaaatcgtgcgtgacatcaaag  R : TGTGTTGGCATAGAGGTCTTTAC | 4 |
| *Angptl4* | F : aagatgcacagcatcacagg  R : atggatgggaaattggagc | 5 |
| *Apoa1* | F : gatgcggtggaacactttct  R : actgtcctcgacttccgaga | 6 |
| *Apoa4* | F : GCACAACAAGCTGGTGCCC  R : CAGGTGCTCCTGCAACTTCTG | 6 |
| *Apob* | F : tgtgtatttgactctgccatca  R : acctcctgaaaatgggactg | 1 |
| *Apoe* | F : ttccgtcatagtgtcctcca  R : cagagctcccaagtcacaca | 1 |
| *Ccl2* | F : AGGTGTCCCAAAGAAGCTGTAG  R : AATGTATGTCTGGACCCATTCC | 7 |
| *Ccl3* | F : TGGAACTGAATGCCTGAGAGT  R : TAGGAGATGGAGCTATGCAGGT | 7 |
| *Ccl5* | F : CCAGAGAAGAAGTGGGTTCAAG  R : AAGCTGGCTAGGACTAGAGCAA | 7 |
| *Cd36* | F : GGCCAAGCTATTGCGACATG  R : CCGAACACAGCGTAGATAGAC | 8 |
| *Chrebp* | F : CTGGGGACCTAAACAGGAGC  R : GAAGCCACCCTATAGCTCCC | 9 |
| *Cpt1a* | F : acagcttccagacgtctctgc  R : atcagtggcctcacagactcc | 1 |
| *Dgat1* | F : GAGGCCTCTCTGCCCCTATG  R : GCCCCTGGACAACACAGACT | 3 |
| *Dgat2* | F : CCGCAAAGGCTTTGTGAAG  R : GGAA TAAGTGGGAACCAGA TCA | 3 |
| *Fabp1* | F :TGAAGGCAATAGGTCTGCCC  R :GTCATGGTCTCCAGTTCGCA | 10 |
| *Fabp2* | F : CATATTCCGTTCCCTGCACT  R : TTGGGGTCTGTGTTCATTCA | 6 |
| *Fasn* | F : gcacctttgatgacatcgtg  R : tcaggtttcagtcccacaga | 1 |
| *Fatp4* | F : gtgagatggcctcagctatc  R : gaagagggtccagatgctct | 11 |
| *Hmgcr* | F : ttggatcgaaggacgaggaaagac  R : gccgagcccacaagattcttgtta | This study |
| *Hmgcs1* | F : tgcagtcttcaatgccgtgaac  R : ctgtaggtctggcatttcctgt | This study |
| *Hmgcs2* | F : TGGTTCAAGACAGGGACACAGAAC  R : AGAGGAATACCAGGGCCCAACAAT | 12 |
| *Il1a* | F : GCCTTATTTCGGGAGTCTAT  R : TAGGGTTTGCTCTTCTCTTACA | 7 |
| *Il1b* | F : GAAGAGCCCATCCTCTGTGA  R : TTCATCTCGGAGCCTGTAGTG | 7 |
| *Il6* | F : CCACGGCCTTCCCTACTTC  R : TCCACGATTTCCCAGAGAACA | 3 |
| *Ldlr* | F : cgtgaacatgactgcaagga  R : tccccactgtgacacttgaa | 1 |
| *Lxra* | F : GCTCTGCTCATTGCCATCAG  R : TGTTGCAGCCTCTCTACTTGGA | 13 |
| *Mttp* | F : acggccattcccattgtg  R : gccagagctccgagagagaa | 6 |
| *Npc1l1* | F : TGGACTGGAAGGACCATTTCC  R : GACAGGTGCCCCGTAGTCA | 14 |
| *Ppara* | F : TCATACATGACATGGAGACCTTG  R : ACTGGCAGCAGTGGAAGAATC | 15 |
| *Ppard* | F : AGATGGTGGCAGAGCTATGACC  R : TCTCCTCCTGTGGCTGTTCC | 16 |
| *Pparg* | F : agctgtcattattctcagtggagac  R : ctgtcttcttgatcacatgcagtag | 17 |
| *Scarb1* | F : acggccagaagccagaagccagtagtc  R : gaccttttgtctgaactccctgtag | 6 |
| *Scd* | F : CCGGAGACCCCTTAGATCGA  R : TAGCCTGTAAAAGATTTCTGCAAACC | 3 |
| *Srebf1* | F : gagccatggattgcacattt  R : cgggaagtcactgtcttggt | 1 |
| *Srebf2* | F : aggtttgtaggttggcagca  R : ccctcagtcaccttctggag | 1 |
| *Tnf* | F : GTCTACTGAACTTCGGGGTGAT  R : ATGATCTGAGTGTGAGGGTCTG | 7 |

**Antibodies**

Primary antibodies used for WB were: anti-SREBP-1 protein (1:500; ab3259, Abcam), anti-S6 ribosomal protein (1:1,000; 2317S, Cell Signaling), anti-phospho-S6 ribosomal protein (S235/S236) (1:1,000; 2211S, Cell Signaling), anti-p70 S6 kinase α (1:200; sc-230, Santa Cruz Biotechnology), anti-Akt (1:1,000; 9272S, Cell Signaling), anti-phospho-Akt (S473) (1:2,000; 4060S, Cell Signaling), anti-phospho-Akt (T308) (1:1,000; 9275S, Cell Signaling), and anti-actin (1:2,000; ab8227, Abcam).

**Supplemental references**

1. Yuan M, Pino E, Wu L, Kacergis M, Soukas AA. 2012. Identification of Akt-independent regulation of hepatic lipogenesis by mammalian target of rapamycin (mTOR) complex 2. J Biol Chem 287:29579–29588.

2. Ding L, Biswas S, Morton RE, Smith JD, Hay N, Byzova TV, Febbraio M, Podrez EA. 2012. Akt3 deficiency in macrophages promotes foam cell formation and atherosclerosis in mice. Cell Metab 15:861–872.

3. Alger HM, Brown JM, Sawyer JK, Kelley KL, Shah R, Wilson MD, Willingham MC, Rudel LL. 2010. Inhibition of acyl-coenzyme A:cholesterol acyltransferase 2 (ACAT2) prevents dietary cholesterol-associated steatosis by enhancing hepatic triglyceride mobilization. J Biol Chem 285:14267–14274.

4. Ikeda K, Mason PJ, Bessler M. 2011. 3’UTR-truncated *Hmga2* cDNA causes MPN-like hematopoiesis by conferring a clonal growth advantage at the level of HSC in mice. Blood 117:5860–5869.

5. Koliwad SK, Kuo T, Shipp LE, Gray NE, Backhed F, So AY-L, Farese RV, Wang J-C. 2009. Angiopoietin-like 4 (ANGPTL4, Fasting-induced Adipose Factor) Is a Direct Glucocorticoid Receptor Target and Participates in Glucocorticoid-regulated Triglyceride Metabolism. J Biol Chem 284:25593–25601.

6. Pan X, Hussain MM. 2009. Clock is important for food and circadian regulation of macronutrient absorption in mice. J Lipid Res 50:1800–1813.

7. Sieber MW, Jaenisch N, Brehm M, Guenther M, Linnartz-Gerlach B, Neumann H, Witte OW, Frahm C. 2013. Attenuated inflammatory response in triggering receptor expressed on myeloid cells 2 (TREM2) knock-out mice following stroke. PloS One 8:e52982.

8. Martin C, Passilly-Degrace P, Gaillard D, Merlin J-F, Chevrot M, Besnard P. 2011. The lipid-sensor candidates CD36 and GPR120 are differentially regulated by dietary lipids in mouse taste buds: impact on spontaneous fat preference. PloS One 6:e24014.

9. Dentin R, Pégorier J-P, Benhamed F, Foufelle F, Ferré P, Fauveau V, Magnuson MA, Girard J, Postic C. 2004. Hepatic Glucokinase Is Required for the Synergistic Action of ChREBP and SREBP-1c on Glycolytic and Lipogenic Gene Expression. J Biol Chem 279:20314–20326.

10. Zhang J-S, Zhang Y-L, Wang H-X, Xia Y-L, Wang L, Jiang Y-N, Li H-H, Liu Y. 2014. Identification of genes related to the early stage of Angiotensin II-induced acute renal injury by microarray and integrated gene network analysis. Cell Physiol Biochem Int J Exp Cell Physiol Biochem Pharmacol 34:1137–1151.

11. Zhan T, Poppelreuther M, Ehehalt R, Füllekrug J. 2012. Overexpressed FATP1, ACSVL4/FATP4 and ACSL1 Increase the cellular fatty acid uptake of 3T3-L1 adipocytes but are localized on intracellular membranes. PLoS ONE 7:e45087.

12. Crawford PA, Crowley JR, Sambandam N, Muegge BD, Costello EK, Hamady M, Knight R, Gordon JI. 2009. Regulation of myocardial ketone body metabolism by the gut microbiota during nutrient deprivation. Proc Natl Acad Sci U S A 106:11276–11281.

13. Schmitt J, Kong B, Stieger B, Tschopp O, Schultze SM, Rau M, Weber A, Müllhaupt B, Guo GL, Geier A. 2015. Protective effects of farnesoid X receptor (FXR) on hepatic lipid accumulation are mediated by hepatic FXR and independent of intestinal FGF15 signal. Liver Int 35:1133–1144.

14. Turley SD, Valasek MA, Repa JJ, Dietschy JM. 2010. Multiple mechanisms limit the accumulation of unesterified cholesterol in the small intestine of mice deficient in both ACAT2 and ABCA1. Am J Physiol Gastrointest Liver Physiol 299:G1012-1022.

15. Kraft CS, LeMoine CMR, Lyons CN, Michaud D, Mueller CR, Moyes CD. 2006. Control of mitochondrial biogenesis during myogenesis. Am J Physiol Cell Physiol 290:C1119-1127.

16. Nahlé Z, Hsieh M, Pietka T, Coburn CT, Grimaldi PA, Zhang MQ, Das D, Abumrad NA. 2008. CD36-dependent regulation of muscle FoxO1 and PDK4 in the PPAR delta/beta-mediated adaptation to metabolic stress. J Biol Chem 283:14317–14326.

17. Voltan S, Martines D, Elli M, Brun P, Longo S, Porzionato A, Macchi V, D’Incà R, Scarpa M, Palù G, Sturniolo GC, Morelli L, Castagliuolo I. 2008. Lactobacillus crispatus M247-derived H_2_O_2_ acts as a signal transducing molecule activating peroxisome proliferator activated receptor-gamma in the intestinal mucosa. Gastroenterology 135:1216–1227.
